# Adaptation of the Freshwater Bloom-Forming Cyanobacterium *Microcystis aeruginosa* to Brackish Water Is Driven by Recent Horizontal Transfer of Sucrose Genes

**DOI:** 10.3389/fmicb.2018.01150

**Published:** 2018-06-05

**Authors:** Yuuhiko Tanabe, Yoshikuni Hodoki, Tomoharu Sano, Kiyoshi Tada, Makoto M. Watanabe

**Affiliations:** ^1^Algae Biomass and Energy System R&D Center, University of Tsukuba, Tsukuba, Japan; ^2^Center for Ecological Research, Kyoto University, Kyoto, Japan; ^3^Center for Environmental Measurement and Analysis, National Institute for Environmental Studies, Tsukuba, Japan

**Keywords:** *Microcystis*, bloom, salt tolerance, sucrose, genomics, brackish water, horizontal gene trasnfer, ecotype

## Abstract

*Microcystis aeruginosa* is a bloom-forming cyanobacterium found in eutrophic water bodies worldwide. *M. aeruginosa* blooms usually occur in freshwater; however, they have also been reported to occur in brackish water. Because *M. aeruginosa* often produces the cyanotoxin microcystin, they are a major concern to public health and environment. Despite this, the ecology, genomic basis, and evolutionary process underlying the *M. aeruginosa* bloom invasion from fresh to brackish water have been poorly investigated. Hence, in the present study, we have sequenced and characterized genomes of two newly discovered salt-tolerant *M. aeruginosa* strains obtained from Japanese brackish water lakes (Lakes Shinji and Tofutsu). Both genomes contain a set of genes for the synthesis of osmolyte sucrose (*sppA, spsA*, and *susA*), hitherto identified in only one strain (PCC 7806) of *M. aeruginosa*. Chemical and gene expression analyses confirmed sucrose accumulation induced by salt. A comprehensive genetic survey of >200 strains indicated that sucrose genes are extremely rare in *M. aeruginosa*. Most surprisingly, comparative genome analyses of the three strains indicated extremely low genetic diversity in the sucrose genes compared with other core genome genes, suggesting very recent acquisitions via horizontal transfer. Invasion of *M. aeruginosa* blooms into brackish water may be a recent event triggered by anthropogenic eutrophication of brackish water.

## Introduction

Water blooms formed by phytoplankton species are often observed in eutrophic bodies of water. Among blooming phytoplankton, *Microcystis aeruginosa* is the most common and widespread cyanobacterial species found in freshwater environments extending from tropical to subfrigid zones (Harke et al., 2016). *M. aeruginosa* blooms cause several environmental problems, including bad odor and bottom-layer hypoxia; however, the problem of greatest concern is the production of hepatotoxic cyanotoxins called microcystins (Harke et al., 2016). Cases of human poisoning (Jochimsen et al., 1998), livestock intoxication (Beasley et al., 1989), and mass mortality of wildlife (Miller et al., 2010a) caused by microcystin contamination have been sporadically reported. Moreover, recent studies suggest increasing frequency of toxic (microcystin-producing) *M. aeruginosa* blooms in response to climate change (Paerl and Otten, 2013).

Most *M. aeruginosa* strains are not well adapted to salinated water (Otsuka et al., 1999; Tonk et al., 2007), so *M. aeruginosa* blooms occur predominantly in freshwater environments. Although less common, *M. aeruginosa* blooms have also been reported in brackish water such as lagoons and estuaries (Preece et al., 2017). Some of these cases can be explained by movement from a freshwater origin (e.g., blooms drifting down a river from a freshwater reservoir to a coastal area) rather than local growth (Miller et al., 2010a). However, the possibility of *M. aeruginosa* blooms in brackish water arising due to genetic acquisition of salt tolerance has not been explicitly studied.

In bacteria, salt tolerance is achieved by a complex mechanism including extracellular transport of various inorganic ions (e.g., Na^+^, K^+^, Ca^2+^, and Cl^−^). However, the hallmark feature of salt-tolerant bacterial cells is the accumulation of low molecular weight osmoprotectant molecules called compatible solutes inside the cells (Le Rudulier et al., 1984). The accumulation of compatible solute decreases the water potential inside the cells so that cells can retain osmotic pressure and avoid dehydration. In addition, physiochemical theory suggests that macromolecules can avoid denaturation under high salinity owing to transformation in water structure induced by compatible solutes (Roberts, 2005). In cyanobacteria, low levels of salt tolerance (up to 0.6 M NaCl) can be achieved by synthesizing and accumulating sugar compatible solutes, sucrose or trehalose, whereas high levels of salt tolerance can be achieved by glucosylglycerol or gluosylglycerate (up to 1.7 M NaCl) and glycine betaine or glutamate betaine (up to 3.0 M NaCl) (Hagemann, 2011). To date, only one strain of *M. aeruginosa, M. aeruginosa* PCC 7806, which was isolated from brackish water in the Netherlands, has been shown to possess genes for compatible solute (Sandrini et al., 2015). PCC 7806 has a low level of salt tolerance [up to 10 g l^−1^ (≈ 0.17 M) of NaCl] (Tonk et al., 2007), and like other lower salt-tolerant cyanobacterial species, it accumulates a sucrose as a compatible solute (Kolman and Salerno, 2016). Three genes have been identified as being responsible for sucrose synthesis in PCC 7806: *sppA, spsA*, and *susA* (Frangeul et al., 2008). Further, molecular and biochemical analyses also suggested that these gene products are responsible for sucrose synthesis and accumulation in PCC 7806 (Kolman et al., 2012; Kolman and Salerno, 2016). However, owing to the lack of genetic information regarding the salt tolerance of *M. aeruginosa*, apart from PCC 7806, whether other salt-tolerant *M. aeruginosa* strains harbor sucrose genes or other genes for salt tolerance remains unknown. The availability of sucrose genes from other strains allows us to infer the tempo and mode of sucrose gene acquisition in *M. aeruginosa*. Such information would provide us with an insight into the bloom occurrence of *M. aeruginosa* in brackish water.

To address these issues, we have characterized two newly discovered salt-tolerant *M. aeruginosa* strains (Sj and NIES-1211) obtained from two different Japanese lakes using whole genome sequencing, growth experiments, and chemical and gene expression analyses. We also demonstrated that salt-tolerant genotypes are extremely rare, whereas their occurrence is widespread at least in Japan. Most surprisingly, comparative genome analyses strongly suggest that the sucrose genes have been acquired by multiple recent horizontal gene transfers (HGT). Our data provide insight into how HGT of genes with an adaptive trait (production of an osmoprotectant) can impact cyanobacterial ecology in aquatic environments.

## Materials and methods

### Strains

**Figure 1:**
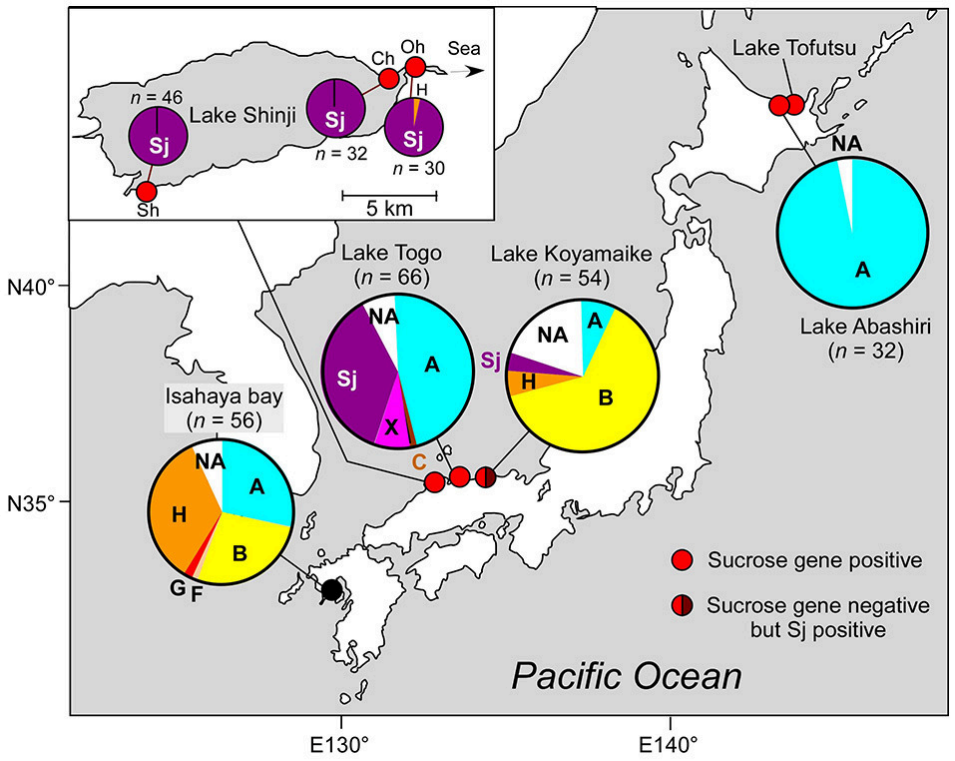
Geographic location and genotypic composition of *M. aeruginosa* in Japanese brackish waters. Different colors in the pie charts indicate clones belonging to different phylogenetic groups (defined in Figure 2). Sj belongs to none of these groups and is indicated in a specific color (purple). The genotypic composition is based on *ftsZ* (Tanabe et al., 2007). Note that group A in Lake Abashiri is represented by a single genotype. Detailed geographic location of sampling sites, sampling dates, and salinities are shown in Supplementary Table S1. *n* indicates number of clones. The data for two locations in Lake Togo are highly similar; therefore, only the data related to Shizen-Koen is shown. Inset: the magnification of Lake Shinji. Sh, Shinji-cho; Oh, Ohashi; Ch, Chidori.

*Microcystis aeruginosa* strain Sj was isolated from a bloom sample at Shinji-cho, Lake Shinji (Figure 1, Supplementary Table S1), using a micropipetting method. The strain will be deposited at the Microbial Culture Collection of the National Institute for Environmental Studies (MCC-NIES, Tsukuba, Japan). Strains NIES-843 and NIES-1211 were obtained from MCC-NIES.

### Growth experiments

A late exponential culture of *M. aeruginosa* in salt-free MA medium (Kasai et al., 2004) was used as an inoculum. Briefly, 500 μl of *M. aeruginosa* cell suspension was inoculated into 10 ml of MA with different final concentrations of NaCl (0, 2.5, 5, 7.5, 10, and 12.5 g l^−1^). Samples were grown in-20 ml screw cap test tubes in an incubation chamber at 23°C under a 12 h light (12–14 μmol photons m^−2^ s^−1^)/12 h dark cycle. Cell concentration was determined by measuring the chlorophyll *a* concentration (Meyer et al., 2016) according to the methanol extraction protocol (Tanabe et al., 2015).

### Phylogenetic analyses

Multilocus sequence typing (MLST) of *M. aeruginosa* Sj was performed according to a published protocol (Tanabe et al., 2007, 2009a). MLST data of other strains used in this study have been previously published (Tanabe and Watanabe, 2011), except for the MLST data of several PCC strains that were retrieved from the whole genome data available from GenBank. Phylogenetic analyses of the seven MLST loci of 250 strains (Supplementary Table S2) were performed by neighbor-joining (NJ) and maximum-likelihood (ML) methods using MEGA version 5.2 (Tamura et al., 2011) and RAxML (Stamatakis, 2006), respectively. The NJ tree reconstruction and bootstrap analysis (1 000 resamplings) employed the maximum composite likelihood substitution model with uniform nucleotide substitution rates among sites and lineages. RAxML was run at Cipres Science Gateway (Miller et al., 2010b) under the default settings, except that bootstrap analysis was performed with 1,000 replicates.

Amino acid alignments of proteins encoding each sucrose gene were generated using clustal X ver. 1.81 (Thompson et al., 1997). The manual removal of gaps and ambiguous sites yielded alignments consisting of 791, 227, and 381 amino acids for *susA, sppA*, and *spsA*, respectively (alignments are available on request). Methods for phylogenetic reconstruction are the same as those for MLST, except that JTT protein substitution model was used for NJ tree reconstruction, and “protein GAMMA” and protein matrix option “AUTO” were used for RAxML analyses.

### Whole genome shotgun analyses

Samples of *M. aeruginosa* Sj and NIES-1211 were not axenic. However, Sj and NIES-1211 cells are much larger than the contaminants, so repeated washing with vacuum filtration using a Mixed Cellulose Esters Membrane (pore size 1.2 μm, Merck Millipore, Carrigtwohill, Ireland) successfully yielded nearly contaminant-free samples. Genomic DNA was extracted from washed Sj using potassium ethyl xanthogenate (Tillett and Neilan, 2000) and from NIES-1211 cells using NucleoBond® AXG Columns with Buffer set III (Macherey-Nagel, Düren, Germany). Whole genome shotgun sequencing of a 350 bp paired-end library constructed using a TrueSeq Nano DNA library Prep Kit (Illumina, San Diego, CA) was performed on a HiSeq 2500 system (Illumina). *De novo* assembly of the contigs was performed using Spades ver. 3.10.1 (Bankevich et al., 2012) and automated annotations were performed using Prokka ver. 1.12 (Seemann, 2014). Contigs of possible contaminants were excluded on the basis of lower coverage (identified by Spades) and a BLAST analysis. The whole genome shotgun datasets of Sj and NIES-1211 have been deposited in the DNA Database of Japan (DDBJ) under accession nos. BDSG01000000 and BEIV01000000, respectively.

### Gene expression analyses

A 1-ml volume of *M. aeruginosa* mid-exponential culture was transferred to a 1.5-ml test tube with or without addition of 10 g l^−1^ NaCl. After 24 h incubation under the same culture condition described in “Growth experiments,” cells were harvested by centrifugation at 20,000 *g* for 10 m, immediately submerged in liquid N_2_, and stored at −80°C until RNA extraction. Salt-free experiments were performed as described above except that a mid-exponential culture of *M. aeruginosa* in MA with 10 g l^−1^ NaCl was centrifuged and resuspended in MA with or without 10 g l^−1^ NaCl. RNA extraction was performed using the RNeasy® Plant Miki Kit (Quiagen, Germantown, MD) according to the manufacturer's protocol with the following minor modification: at the initial step, zirconium beads (0.1 mm in diameter) were added into the microtube and cells were disrupted using a mini Bead-Beater (Biospec Products, Bartlesville, OK) at 4,200 rpm for 30 s. Residual DNA was dissolved using the RNase-Free DNase Set (Quiagen). Contamination of genomic DNA was checked by PCR reactions using the primer pair ftsF/ftsR targeting *ftsZ* DNA (Tanabe et al., 2007). cDNA synthesis and RT-qPCR reactions were performed using the One Step SYBR® PrimeScript™ PLUS RT-PCR kit (Takara, Shiga, Japan) with sucrose gene specific primers (Supplementary Table S3) according to the manufacture's protocol. Transcripts of *rnpB* were used as a reference (Makower et al., 2015). The primer specificities for RT-qPCR reactions were confirmed by melting curve analysis (Ririe et al., 1997). The results of RT-qPCR were assessed using the comparative C_T_ method (Schmittgen and Livak, 2008). All RT-qPCR analyses were performed using the Applied Biosystems StepOnePlus Real-Time PCR system (Thermo Fisher Scientific, Waltham, MA).

### Sucrose gene detection and genotyping

Detection of three sucrose genes (*sppA, spsA*, and *susA*) was performed using the primer pairs, sppA_1F/1R, spsA_1F/1R, and susA_1F/1R, respectively (Supplementary Table S4). The genome sequencing of Sj revealed that these primers did not cover polymorphic sites found between Sj and PCC 7806 strains. Therefore, two new primer pairs, spsA_3F/3R and susA_2F/2R, were used for PCR genotyping of *spsA* and *susA*. PCR conditions and sequencing methods were the same as those reported for MLST loci (Tanabe et al., 2007) except for the annealing temperatures (Supplementary Table S4).

### Genetic composition analyses

Genetic composition analysis of *M. aeruginosa* using clone library analyses was based on *ftsZ*, one of the seven MLST loci (Tanabe et al., 2007). This locus can be used to assign the *ftsZ* genotype into defined MLST phylogenetic groups (Tanabe and Watanabe, 2011). Environmental DNA was extracted using a FastDNA SPIN kit (MP Biomedicals, Tokyo, Japan). PCR amplification was performed using the high fidelity DNA polymerase *Pyrobest*® (Takara, Shiga, Japan). Amplified fragments were cloned using the Zero Blunt® TOPO® PCR cloning kit (Thermo Fisher Scientific) and >30 *ftsZ*-positive clones were sequenced. A clone was assigned to a specific group when the sequence was of the same genotype as that assigned by previous MLST (Supplementary Table S2) or located inside the group defined by MLST. Otherwise, the clone was designated “NA.” All *ftsZ* sequence data are available as Supplementary Data S1.

### Sucrose analyses

Mid- to late-log phase precultures of Sj were filtrated gently using an Isopore^TM^ membrane filter (pore size 1.2 μm, Merck Millipore) to minimize bacterial contamination and possible bacterial sucrose synthesis, and then resuspended at 5 ml Sj cell suspension to 5.5 ml fresh MA medium with or without 10 g l^−1^ NaCl in 20-ml test tubes. Tubes were incubated in the chamber described above for 24 h. Before sucrose extraction, cultures were again filtrated using the Isopore™ membrane filter (pore size 1.2 μm) to minimize bacterial contamination. Cells on the filter were resuspended in MA medium. A 300-μl aliquot of the suspension was used for chlorophyll extraction and measurement, and the remaining suspension was filtered using a Durapore® membrane filter (pore size 0.65 μm, Merck Millipore). The filter with Sj cells was soaked in 7 ml of 80% MeOH and the supernatant subjected to sucrose extraction (Ehira et al., 2014). Sucrose concentration was measured by high-performance liquid chromatography using a chromatograph equipped with a SUGAR SP0810 column (8 × 300 mm; Shodex, Tokyo, Japan) maintained at 80°C and a refractive index detector, with deionized water as the eluent at a flow rate of 1 ml min^−1^ and a sample volume of 20 μl. Sucrose content was normalized to chlorophyll *a* concentration as in previous study (Ehira et al., 2014).

### Microcystin analyses

A 5-ml sample of exponential phase Sj culture was mixed with 0.25 ml of acetic acid, sonicated for 10 min, and then centrifuged at 3000 rpm for 20 min (step one). To the pellet, 0.5 ml of methanol was added and the mixture was sonicated and centrifuged (step two). To the combined supernatants from steps one and two, 5 ng of ^15^N labeled microcystin variants (microcystin-LR, -RR, -WR, -FR, -YR, and−7 desmethyl LR) was added as surrogates (Sano et al., 2011). The mixture was absorbed onto a HLB 1 cc cartridge (Waters, Milford, MA), washed with 20% methanol, and finally eluted with 0.5 ml of 90% methanol. The cartridge was then rinsed with 0.5 ml dH_2_O. Microcystins were analyzed using 20 μl of the combined eluates by LC–MS/MS (LCMS-8040, Shimadzu, Kyoto, Japan). Each microcystin concentration was determined using calibration curves generated by measuring a series of microcystin standards of the variants listed above. Microcystin content was normalized using chlorophyll *a* concentration as in a previous study (Meyer et al., 2016).

## Results and dicussion

### Genomic and physiological features of salt-tolerant strain *M. aeruginosa* Sj

**Figure 2:**
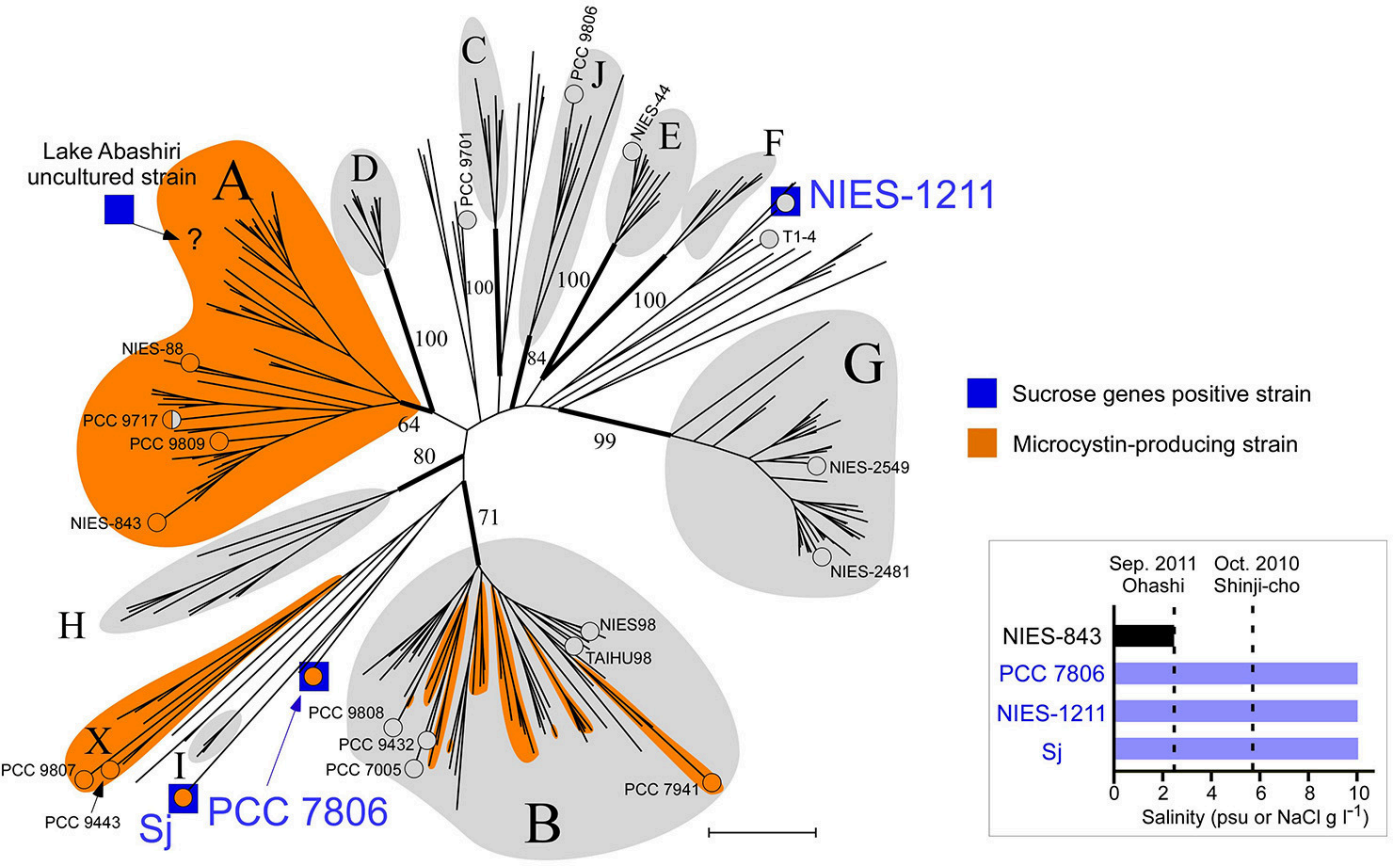
Phylogeny of salt-tolerant *M. aeruginosa*. A multilocus neighbor-joining (NJ) phylogenetic tree based on seven housekeeping loci (Tanabe et al., 2007). Groups are defined according to a previous report (Tanabe and Watanabe, 2011) with three new designations (groups H, I, and J). Numbers at the selected nodes indicate bootstrap values inferred from RaxML. *M. aeruginosa* strains for which a whole genome sequence was available are indicated by “°” and the strain name. Strain PCC 9717 is indicated in half-orange color because it has an incomplete microcystin synthetase gene cluster (Humbert et al., 2013). A possible placement of a Lake Abashiri clone (positive for sucrose genes) in group A is indicated with “?.” Scale bar, 0.005 substitutions per sites. Inset: Salt tolerance of three sucrose gene-positive strains and one sucrose gene-negative strain. Note that psu (salt concentration determined from conductivity) and NaCl concentration in MA medium are substantially the same value.

First, we analyzed *M. aeruginosa* strain Sj, which has recently been isolated from a Japanese eutrophic brackish water lake, Lake Shinji (Figure 1). Lake Shinji is connected to the sea by a river and another brackish water lake (Lake Nakaumi), and its salinity fluctuates from 0.5 to 8 psu (practical salinity unit) depending on meteorological conditions (Uye et al., 2000). Since 1964, *M. aeruginosa* blooms have been reported occasionally in the lake. In the year in which Sj was isolated, the *M. aeruginosa* bloom was very dense (Supplementary Figure S1), reaching >89 μg chlorophyll *a* l^−1^. Genetic composition analyses of *M. aeruginosa* on the basis of *ftsZ* indicated that the *M. aeruginosa* blooms in 2010 and 2011 were genetically highly homogenous, comprising almost entirely of a single genotype, which was the same as that of Sj (Figure 1). The salinity of Lake Shinji from which samples were collected for analyzing genetic composition was 2.5–5.8 psu (Supplementary Table S1), markedly higher than the salinity tolerance level of most known *M. aeruginosa* strains (Otsuka et al., 1999; Tonk et al., 2007). These observations suggest that the Sj genotype was adapted to high salinity as early as 2010 or 2011. Indeed, strain Sj showed salt tolerance up to 10 g l^−1^ NaCl, higher than the salinity of the lake when the strain was isolated (Figure 2 and Supplementary Figure S2A). The observed level of salt tolerance is comparable to other salt-tolerant *M. aeruginosa* strains or conspecific cultures (Orr et al., 2004; Tonk et al., 2007). Even higher salt tolerance, up to 17 g l^−1^ NaCl, was obtained in previous reports (Robson and Hamilton, 2003; Tonk et al., 2007). The salinity tolerance of Sj did not exhibit any improvement even when salt-acclimated cells were used as seeds for the growth experiment (Supplementary Figure S2B), suggesting the presence of stable genetic factors conferring the observed level of salt tolerance.

**Figure 3:**
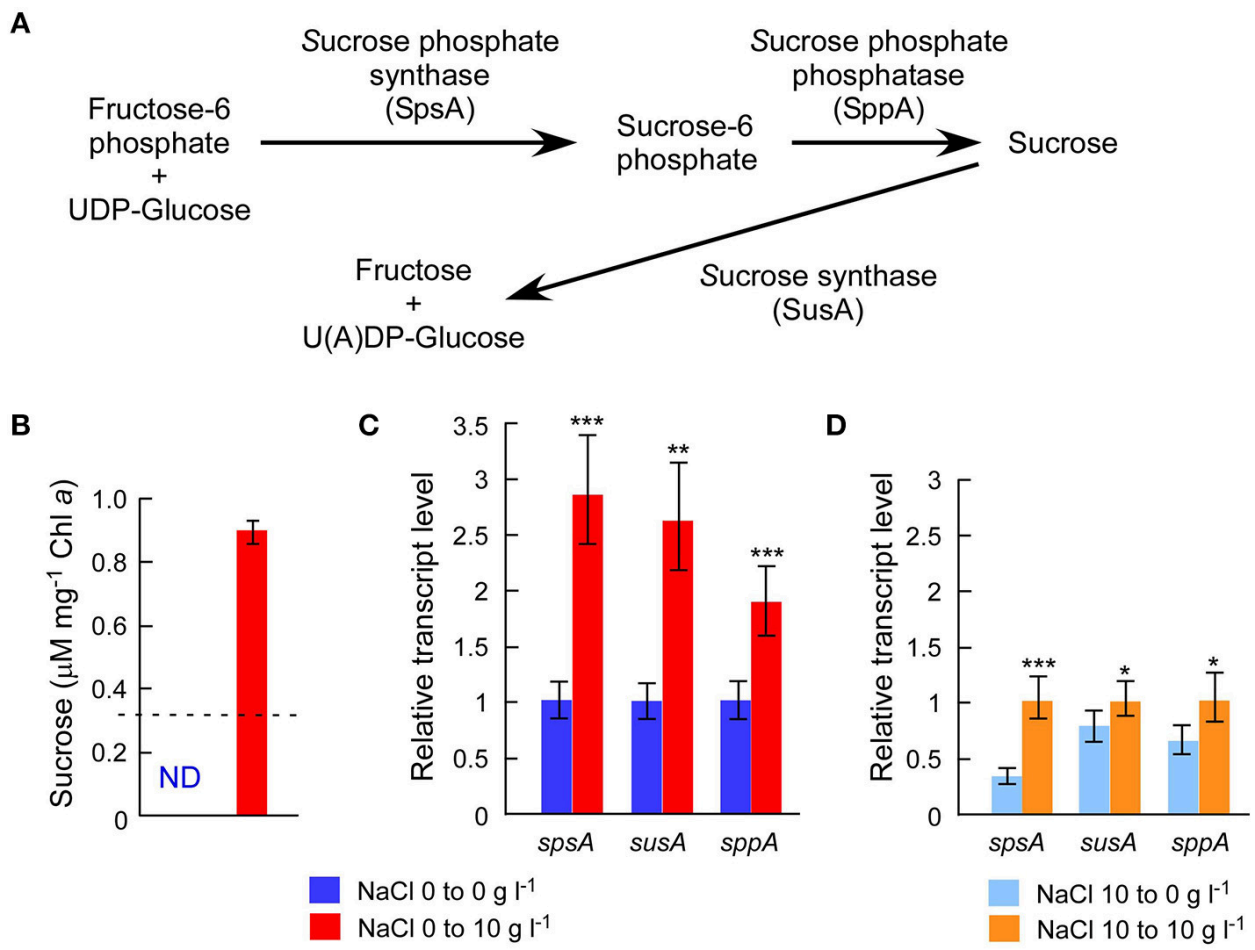
Chemical analysis of intracellular sucrose and sucrose gene expression analyses. **(A)**, Sucrose gene products and their contributions to sucrose metabolism in *M. aeruginosa*. **(B)**, Sucrose concentrations after 24 h incubation with or without 10 g l^−1^ NaCl. The dotted line indicates the detection limit of liquid chromatography. The sucrose concentration was >3 times higher in salt-treated cells than controls. **(C)**, Results of RT-qPCR after the same treatment in **(B)**. **(D)**, Results of RT-qPCR after 24 h incubation with or without 10 g l^−1^ NaCl using salt-acclimated cells as a seed. Bars indicate 95 % confidence intervals calculated from three biological and three technical replicates (by Student's t, df = 16). Statistical values are the results of homoscedastic one-tailed *t*-tests of ΔC_T_ means: ^*^P < 0.05; ^**^P < 0.01; ^***^P < 0.001.

The draft genome sequence of Sj identified a set of genes (*sppA, spsA*, and *susA*) for compatible solute sucrose synthesis (Figure 3A), while none of the genes for other compatible solutes were found in the genome (Supplementary Table S5). Chemical analysis also revealed sucrose accumulation in response to salt (Figure 3B). Consistent with sucrose accumulation as an osmoprotectant mechanism in salt-tolerant cyanobacteria, all three sucrose genes were expressed in Sj, and expression levels were upregulated in response to salt (Figures 3C,D). In contrast, the expression of the sucrose synthase gene *susA* was only slightly downregulated at 24 h after the transfer of Sj from saline to salt-free media (Figure 3D). This observation is consistent with previous findings that the *susA* product is involved in sucrose breakdown (Figure 3A) rather than synthesis (Curatti et al., 2002; Hagemann, 2011). It is likely that *susA* expression is sustained until the intracellular sucrose concentration is reduced to the level of the non-saline condition. On the other hand, the strain NIES-843, which does not possess these sucrose genes (Kaneko et al., 2007), showed much lower salt tolerance (Figure 2 and Supplementary Figure S2C).

A previous study detected another compatible solute, trehalose, in *M. aeruginosa* PCC 7806 (Meissner et al., 2015), although no homologs of known genes for trehalose synthesis were found in the PCC 7806 genome (Klähn and Hagemann, 2011). Our LC analysis did not detect trehalose in *M. aeruginosa* Sj, consistent with the absence of genes for trehalose synthesis in the Sj genome (Supplementary Table S5). However, it is possible that the trehalose concentration in Sj is below the detection limit in this study. The identification of genes responsible for trehalose synthesis in PCC 7806 is critical to clarify this issue.

### Genomic and physiological features of salt-tolerant strain *M. aeruginosa* NIES-1211

**Figure 4:**
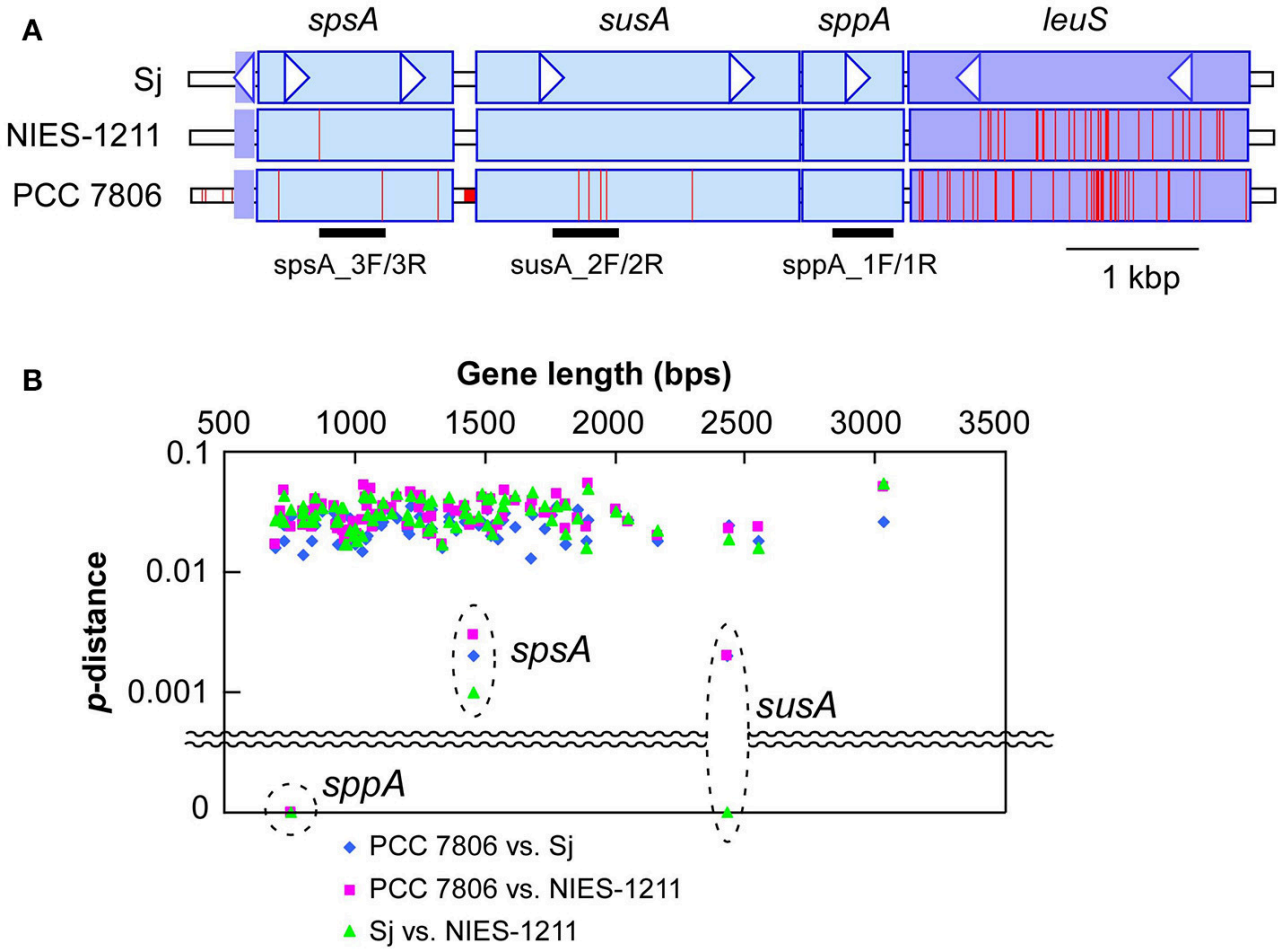
Genetic diversity of sucrose genes. **(A)**, DNA polymorphisms within sucrose genes, and the adjacent *leuS* locus encoding leucyl-tRNA synthetase. A nucleotide site different from that of Sj is indicated by a red bar. PCR targets for sucrose genotyping are indicated below the gene diagram. Genes located downstream of *leuS* showed a level of genetic diversity similar to that shown by *leuS* (Supplementary Figure S6). **(B)**, Genetic distances of sucrose genes and selected core genome genes (>650 bps in length) including the seven MLST loci (Tanabe et al., 2007) and *leuS*. The core genome genes included in this analysis are listed in Supplementary Table S6. X-axis indicates nucleotide length of the genes. Y-axis indicates percent nucleotide difference (*p*-distance) on a log scale.

Sucrose genes are extremely rare in *M. aeruginosa*. Of 20 *M. aeruginosa* strains for which whole genome sequence data are currently available, only PCC 7806 possesses these sucrose synthesis genes in addition to Sj (Supplementary Table S2). A comprehensive PCR survey of 210 available strains found none with sucrose genes except NIES-1211 (Supplementary Table S2), which is also of brackish water origin (from Lake Tofutsu, Figure 1). Although the salinity of Lake Tofutsu was not documented at the time of isolation, NIES-1211 showed salt tolerance up to 10 g l^−1^ (Figure 2 and Supplementary Figures S2D,E). We sequenced the whole genome of NIES-1211 to investigate its genomic basis for salt tolerance. The draft genome of NIES-1211 also revealed the presence of the same three sucrose synthesis genes as those of Sj and PCC 7806 (Figure 4A), while none of the genes for other compatible solutes were found in the genome (Supplementary Table S5). In addition, similar to Sj, the expression levels of these sucrose synthesis genes were upregulated in NIES-1211 by salt (Supplementary Figure S3).

Of note, none of the other genes possibly involved in salt acclimation (Guljamow et al., 2007; Sandrini et al., 2015) are exclusively shared among salt-tolerant *M. aeruginosa* strains (Supplementary Table S5). For example, the genes *actM* and *pfnM*, which were previously suggested to have a possible involvement in salt tolerance (Guljamow et al., 2007), are absent in Sj and NIES-1211. These results suggest that sucrose genes are responsible for the salt tolerance of *M. aeruginosa*, and thus allow for *M. aeruginosa* blooms in brackish water up to 10 psu.

### Microcystin content in response to salt stress

The genome of Sj contains the complete microcystin synthetase gene cluster (*mcyABCDEFGHIJ*), which is responsible for the biosynthesis of microcystins (Tillett et al., 2000), whereas the NIES-1211 genome contains none of the *mcy* genes. Microcystins are cyclic heptapeptides produced nonribosomally (Tillett et al., 2000) in many *M. aeruginosa* strains (Figure 2 and Supplementary Table S2). The biological role of microcystins *in M. aeruginosa* is a matter of long-standing debate (Kaplan et al., 2012). Recently, a study reported that microcystins protect cells from oxidative stress by binding and stabilizing proteins, including the RuBisCo enzymes (Zilliges et al., 2011). In this context, whether salt stress affects the level of microcystins in Sj cells as in the response to oxidative stress is of particular interest for both the ecology and environmental impact of *M. aeruginosa*. To test this, we performed quantitative analyses of microcystins and their gene expressions in Sj. Both microcystin synthetase genes (*mcyA* and *mcyE*) expressions and chemical microcystin quantification analyses of Sj indicated that salinity did not significantly affect microcystin concentration per cell throughout the growth period (Supplementary Figure S4). This suggests that microcystins do not respond to salt stress, unlike oxidative stressors, at least in Sj.

### Sucrose gene acquisition via recent HGT

**Figure 5:**
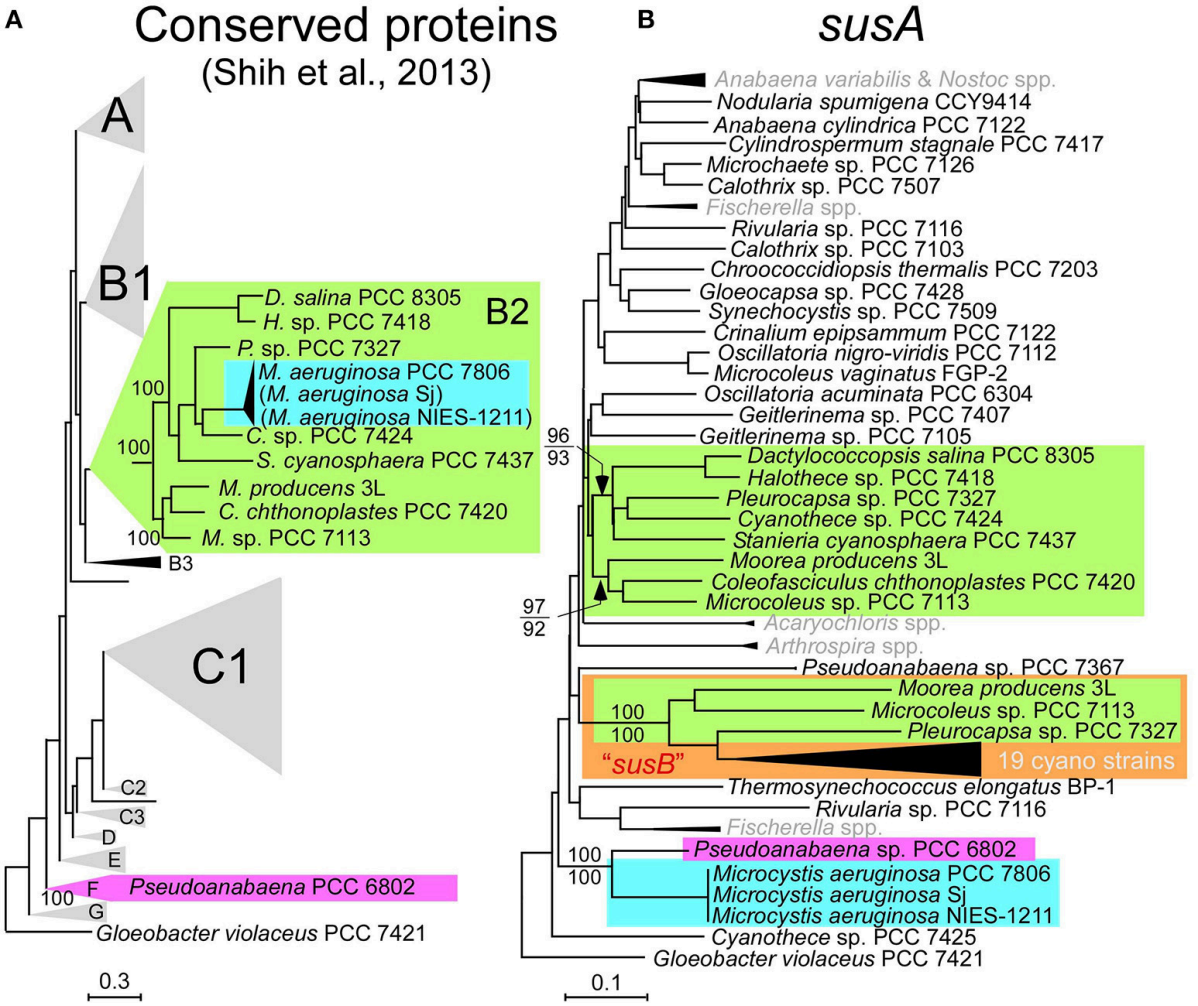
Phylogenetic trees. **(A)**, The phylogenetic tree modified from the conserved protein-based tree with the group designations by Shih et al. (2013). *M. aeruginosa* Sj and NIES-1211 are not included in the analysis. However, whole-genome blast analyses indicated that Sj and NIES-1211 are most closely related to PCC 7806 in the tree (indicated in parentheses). **(B)**, A NJ phylogenetic tree of *susA*. Bootstrap values (>70) on the basis of 1 000 replicates are indicated at the respective nodes. Numbers in parentheses after the strain name indicate GenBank protein IDs. The color-coding in **(B)** is according to **(A)**. *susB* (a homolog of *susA*; Kolman et al., 2012) is highlighted by the orange box. OTUs in gray in **(B)** are compressed. Scale bar, substitutions per sites.

Multilocus phylogenetic analyses indicated that sucrose synthesis is sporadic in *M. aeruginosa* (Figure 2). Given the frequent horizontal gene transfer (HGT) in *M. aeruginosa* (Guljamow et al., 2007; Tanabe et al., 2009b; Humbert et al., 2013), this rare and patchy distribution of salt tolerance genes is not surprising. Indeed, it was proposed that *M. aeruginosa* likely acquired these sucrose genes by HGT from a distantly related cyanobacterium (Kolman and Salerno, 2016). A comparison of the genome (Shih et al., 2013) and sucrose gene phylogenetic trees of diverse cyanobacteria including Sj and NIES-1211 also support HGT rather than vertical transmission of sucrose genes from distantly related cyanobacteria (Figure 5). However, the extremely low genetic diversity of sucrose genes among the three salt-tolerant strains is highly unexpected (Figure 4A). Further the core genome genes including the adjacent *leuS* locus show substantial genetic distance among the three strains regardless of individual gene length (Figure 4B). For example, the percent divergence of the three sucrose genes, *sppA, spsA*, and *susA* between Sj and NIES-1211 were 0, 0.1, and 0, respectively; these orders of magnitude are lower than those of other genes in the genome (percent divergence, >1). This discrepancy can most likely be explained by the very recent introgression of sucrose genes into *M. aeruginosa* and subsequent spread across different strains of *M. aeruginosa*. However, multiple acquisitions from the same source can not be strictly ruled out. The pattern of genetic diversity and the distribution of sucrose genes in the MLST phylogenetic tree suggest either one or two HGTs have occurred within *M. aeruginosa*. It is evident that NIES-1211 has independently acquired sucrose genes via HGT. However, the possiblity of the vertical transmission of sucrose genes in Sj and PCC 7806 is obscured by the extremely low genetic diversity between the two strains. Thus, Sj and PCC 7806 may have independently acquired sucrose genes via HGT. In *susA* and *sppA* trees (Figure 5B and Supplementary Figure S5A), the sequences of *M. aeruginosa* are most closely related to those of *Pseudoanabaena* sp. PCC 6802, whereas the sequence of *spsA* of *M. aeruginosa* is closely related to those of nitrogen fixing cyanobacteria, including *Cyanothece* sp. PCC 7425 (Supplementary Figure S5B). This phylogenetic discordance suggests that the sucrose gene cluster in *M. aeruginosa* is a composite of genes from different cyanobacteria. Given the high sequence similarity of the sucrose gene cluster among strains of *M. aeruginosa*, the build-up of the gene cluster would predate the time of its import into *M. aeruginosa*. In any event, the mosaic nature of the gene cluster reflects a complex evolutionary history of the sucrose gene cluster in cyanobacteria

**Figure 6:**
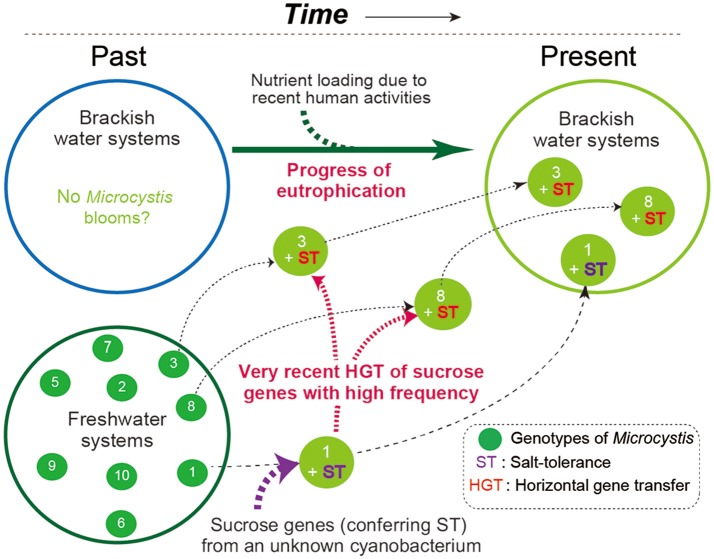
A schematic of the hypothesized *M. aeruginosa* bloom occurrence in Japanese brackish water. In the past, *M. aeruginosa* blooms were restricted to freshwater systems because *M. aeruginosa* is salt-sensitive. At some point in time, one *M. aeruginosa* genotype became salt-tolerant owing to the acquisition of sucrose genes via HGT. Recent eutrophication of brackish water systems caused by human activity has driven the subsequent spread of the sucrose genes in different genotypes of *M. aeruginosa*. As a result, salt-tolerant *M. aeruginosa* have become prevalent and can form blooms in brackish water systems. Note that different numbers indicate different genotypes.

Field surveys of several Japanese brackish water lakes revealed widespread occurrence of the same sucrose gene genotype as Sj in brackish water with salinity >1.3 psu (Supplementary Table S1). The sucrose genes identified in Lake Togo can be ascribed to the presence of Sj or a similar strain (Figure 1). However, the same sucrose genes were also identified in another brackish water lake, Lake Abashiri, in which Sj has not been observed (Figure 1 and Supplementary Table S1). The prevalence of a single *ftsZ* genotype belonging to group A (Figure 2), which is only distantly related to Sj, might be an evidence of another independent and very recent HGT of sucrose genes. Collectively, these results suggest a very recent expansion of sucrose gene expression and salt tolerance to *M. aeruginosa* in Japanese brackish water environments (Figure 6).

### Ecological and evolutionary implication

It is currently unclear why the observed sucrose gene HGT among *M. aeruginosa* has such a short evolutionary history. One hypothesis is that eutrophication of brackish water is a prerequisite for the emergence of salt-tolerant *M. aeruginosa* and its invasion from fresh water. Increasing nutrient loading by human activity around coastal areas including lagoons may be the driving force for the recent emergence of salt tolerance in *M. aeruginosa* via HGT. In this scenario, the salt-tolerant *M. aeruginosa* strains identified in this study represent new and potentially stable and long-lasting ecotypes (Cohan, 2002). Another possibility is that the emergence of salt-tolerant *M. aeruginosa* strains in brackish water is transient. In this scenario, while recurrent HGTs of sucrose genes would give rise to salt-tolerant *M. aeruginosa* under favorable salinity conditions, salt tolerance is adaptive in any one place for a very short time owing to salinity fluctuation, such that this new ecotype would immediately become extinct, as reflected in the “species-less model” (Cohan, 2011). We favor the former possibility, however, because *M. aeruginosa* blooms are considered relatively recent phenomena in response to anthropogenic nitrogen loading from agricultural, domestic, and industrial effluents (Harke et al., 2016). In fact, a stable isotope study suggested that the eutrophication in Lake Shinji began in the 1940's (Yamamuro and Kanai, 2005). Nutrient-rich brackish water would favor the mass proliferation of non-N_2_ fixing cyanobacteria like *M. aeruginosa* with salt tolerance rather than other salt-tolerant N_2_-fixer species (Harke et al., 2016). In either case, these salt-tolerant *M. aeruginosa* genotypes appear to have been generated very recently.

## Conclusions

Our data clearly shows that at least a few instances of *M. aeruginosa* bloom in brackish water were caused by salt-tolerant genotypes harboring sucrose genes. We concluded that transfer of sucrose genes among *M. aeruginosa* is an occurrence with a short evolutionary history; however, the possibility of independent introgression into each strain from unknown vectors (e.g., cyanophages) harboring the same genes cannot be ruled out. We hypothesize that the recent HGT coincided with recent anthropogenic eutrophication in brackish water bodies. Further investigations on the origin of sucrose genes in *M. aeruginosa* and worldwide field surveys for identification of other salt-tolerant genotypes would help clarify how the rapid evolution of salt-resistance occurred in this ubiquitous toxin-producing cyanobacterial species.

## Author contributions

YT designed the research, YT and YH collected samples, YT performed growth experiments, DNA and gene expression analyses, whole genome analyses, and phylogenetic analyses, YT, KT, and MW performed sucrose analyses, TS performed microcystin analyses, and YT, YH, TS, and KT wrote the paper. All authors read and approved the final manuscript.

### Conflict of interest statement

The authors declare that the research was conducted in the absence of any commercial or financial relationships that could be construed as a potential conflict of interest.
