# Supplementary material for: Adaptation of the Freshwater Bloom-Forming Cyanobacterium Microcystis aeruginosa to Brackish Water Is Driven by Recent Horizontal Transfer of Sucrose Genes
Source: Front Microbiol. 2018 Jun 5;9:1150. doi: 10.3389/fmicb.2018.01150 (PMC5996124; doi:10.3389/fmicb.2018.01150)
Supplement: Supplementary file 1 [file Table_1.PDF]

Supplementary Table S1. Environmental water samples used for the clone library and sucrose gene detection analyses.

Locality	Latitude/Longitude	dd/mm/yy	Salinity [psu]	*M. aeruginosa* [cell ml^{-1}]	Sj[b]	Sucrose genes[a] *spsA*	*sppA*	*susA*
Chidori, Lake Shinji	N35° 27’ 55", E133° 02’ 43’’	19/10/10	4.6	2.9 x 10^3	+	Sj	Sj	Sj
Shinji-cho, Lake Shinji	N35° 24’ 44", E132° 55’ 19’’	19/10/10	5.8	5.7 x 10^3	+	Sj	Sj	Sj
Ohashi, Lake Shinji	N35° 28’ 02.43", E133° 03’ 19.37’’	25/09/11	2.5[c]	+[d]	+	Sj	Sj	Sj
Lake Abashiri	N43° 59’ 36.67", E144° 13’ 19.15’’	01/08/16	1.5	+[d]	-	Sj	Sj	Sj
Shizen-Koen, Lake Togo	N35° 29’ 00.96", E133° 52’ 56.09’’	11/10/16	1.3	<10[e]	+	Sj	Sj	Sj
Hawaii, Lake Togo	N35° 29’ 28.14", E133° 53’ 21.69’’	11/10/16	1.5	<10[e]	+	Sj	Sj	Sj
Lake Koyamaike	N35° 30’ 11.45", E134° 10’ 18.42’’	11/10/16	4.6	<10[e]	+	-[f]	-[f]	-[f]
Isahaya Bay reservoir	N32° 52’ 35.01", E130° 10’ 04.80’’	30/10/16	0.07	+[d]	-	-	-	-

[a] Results of PCR detection and genotyping. Sj indicates the same genotype as that of Sj.

[b] Detection of Sj genotype by *ftsZ* -based clone library analysis.

[c] Data from the same location 12 days earlier, retrieved from http://www.cgr.mlit.go.jp/izumokasen/ (in Japanese).

[d] Presence of *M. aeruginosa* was confirmed under a microscope, but not enumerated.

[e] Not detected in 10 µl of 10x concentrated water sample under a microscope, indicating < 10 cells per 1 ml.

[f] Negative results are likely due to low biomass concentration of Sj genotype.
