# Supplementary material for: Adaptation of the Freshwater Bloom-Forming Cyanobacterium Microcystis aeruginosa to Brackish Water Is Driven by Recent Horizontal Transfer of Sucrose Genes
Source: Front Microbiol. 2018 Jun 5;9:1150. doi: 10.3389/fmicb.2018.01150 (PMC5996124; doi:10.3389/fmicb.2018.01150)
Supplement: Supplementary file 2 [file Table_2.PDF]

Supplementary Table S2. Strain information.

ST[a]	Representative strain	Allele numbers[b]							*mcyG* [c]	Microcystins[d]	Sucrose genes[e]	Group[f]
		ftsZ	*glnA*	*gltX*	*gyrB*	*pgi*	*recA*	*tpi*				
**3**	NIES-88[g]	3	3	3	3	3	3	3	+	+	-	A
**4**	NIES-89	4	4	4	4	4	4	4	+	+	-	A
**5**	NIES-90	5	5	5	5	5	5	5	+	+	-	A
**10**	NIES-102	9	9	8	8	9	8	8	+	+	-	A
**13**	NIES-107	5	5	5	10	5	11	10	+	+	-	A
**18**	NIES-843[g]	9	15	15	12	16	14	13	+	+	-	A
**19**	CL4	9	15	15	12	16	14	8	+	+	-	A
**20**	MCS3	13	16	16	13	17	3	14	+	+	-	A
**22**	NIES-902 (=TL2)	15	18	18	5	19	11	16	+	+	NA	A
**26**	NIES-103	9	15	21	12	16	8	8	+	+	-	A
**28**	NIES-1085 (=TAC86)	17	20	23	16	23	18	20	+	+	-	A
**29**	NIES-1063 (=TAC60)	18	21	3	17	3	14	21	+	+	-	A
**32**	NIES-1209 (=TAC350)	5	24	5	10	5	21	5	+	+	-	A
**43**	NIES-1108 (=TAC129)	5	5	34	26	5	28	30	+	+	-	A
**49**	NIES-1150 (=TAC177)	30	15	23	31	37	34	34	+	+	-	A
**52**	NIES-1114 (=TAC135)	5	5	5	10	5	11	36	+	+	-	A
**55**	NIES-1139 (=TAC165)	31	41	3	3	3	35	37	+	-	-	A
**55**	NIES-1141 (=TAC167)	31	41	3	3	3	35	37	+	+	-	A
**66**	NIES-1102 (=TAC122)	9	47	8	12	9	8	8	+	+	-	A
**72**	NIES-1234 (=TAC378)	36	18	50	39	5	46	43	+	+	-	A
**76**	NIES-1131 (=TAC155)	39	54	54	43	49	14	47	+	+	-	A
**80**	NIES-2465 (=KA3b)	39	56	58	45	51	50	48	+	+	-	A
**81**	NIES-2479 (=KA4)	41	18	59	5	5	16	49	+	+	-	A
**82**	NIES-2466 (=KA6)	5	5	5	26	5	11	50	+	+	-	A
**84**	NIES-2467 (=SA2)	39	58	61	43	53	51	51	+	+	-	A
**85**	NIES-2468 (=Sw4)	9	15	15	46	16	8	8	+	+	-	A
**87**	NIES-2474 (=Ks05TA62)	39	47	54	43	49	34	48	+	+	-	A
**90**	NIES-2469 (=Ks05IS02)	4	61	4	43	4	8	8	+	+	-	A
**92**	Ks05YA11	39	56	58	50	51	55	48	+	+	-	A

**93**	Tn05AK01	42	16	65	13	56	56	52	+	+	-	A
**94**	NIES-2475 (=Tn05AK02)	43	4	66	43	4	8	4	+	+	-	A
**96**	NIES-2476 (=Tn05AK05)	39	56	68	43	57	55	48	+	+	-	A
**97**	NIES-2561 (=In05Yo05)	44	15	69	50	58	8	54	+	+	-	A
**98**	NIES-2477 (=Ia05Yo03)	30	15	5	50	59	38	55	+	+	-	A
**99**	Ia05Yo05	30	15	23	50	59	38	55	+	+	-	A
**100**	NIES-2478 (=Sn05Mb05)	36	18	70	26	5	46	56	+	+	-	A
**101**	LNN-s1	15	18	18	5	5	11	57	+	+	-	A
**106**	SA1	39	58	61	43	53	11	51	+	+	-	A
**108**	SHA4	43	4	66	12	4	18	58	+	+	-	A
**163**	Sn05Mb06	36	18	50	10	5	77	30	+	+	-	A
**185**	NIES-2598 (=Kn07TS91)	30	15	23	84	91	38	34	+	+	-	A
**188**	NIES-1174	30	15	23	87	94	89	34	+	+	NA	A
**194**	NIES-1185	36	18	50	26	5	46	78	+	+	NA	A
**198**	Sn07Hb02	39	111	114	89	98	34	79	+	+	-	A
**202**	Bs07BH02	30	15	23	50	59	38	81	+	+	-	A
**203**	NIES-2604 (=Bv07BH03)	4	4	4	4	4	92	4	+	+	-	A
**204**	Ys07BZ04	36	5	117	26	5	46	30	+	+	-	A
**214**	Ka08TS03	43	4	21	93	4	18	8	+	+	-	A
**229**	Aw08Gb01	66	123	129	13	108	98	47	+	+	-	A
**230**	NIES-2610 (=An08Hj03)	66	124	129	13	109	99	47	+	+	-	A
**234**	NIES-2611 (=As08Fu06)	66	124	129	13	109	100	47	+	+	-	A
**243**	PCC 9717[g]	5	132	23	50	117	106	48	+[h]	NA	-	A
**247**	PCC 9809[g]	66	4	141	13	4	8	48	+	+	-	A
**6**	NIES-98[g]	6	6	6	6	6	6	6	-	-	-	B
**7**	NIES-99	6	7	6	6	6	6	6	-	-	-	B
**9**	NIES-101	8	7	6	6	8	6	6	-	-	-	B
**21**	PCC 7941[g]	14	17	17	14	18	6	15	+	+	-	B
**31**	NIES-1105 (=TAC125)	20	23	25	19	25	20	6	+	+	-	B
**33**	NIES-1238 (=TAC382)	6	25	26	20	8	17	6	+	+	-	B
**35**	NIES-1069 (=TAC67)	22	27	28	20	8	23	6	+	+	-	B
**36**	NIES-1070 (=TAC69)	6	28	28	20	6	23	24	+	+	-	B

**37**	NIES-1077 (=TAC76)	23	29	29	22	27	24	25	-	-	-	B
**42**	NIES-1107 (=TAC128)	26	32	33	25	32	27	29	-	-	-	B
**44**	NIES-1115 (=TAC136)	27	33	26	27	33	29	6	-	-	-	B
**45**	NIES-1122 (=TAC146)	26	34	33	28	32	30	31	-	-	-	B
**48**	NIES-1134 (=TAC159)	20	37	36	8	36	33	24	+	-	-	B
**51**	NIES-1113 (=TAC134)	20	38	38	20	36	20	35	+	-	-	B
**60**	NIES-298[g]	6	44	42	35	42	23	6	+	+	-	B
**61**	NIES-299	20	45	43	36	33	39	6	-	-	-	B
**62**	NIES-478	14	17	44	36	43	40	6	+	+	-	B
**63**	NIES-1075 (=TAC74)	20	46	45	37	33	24	6	-	-	-	B
**65**	NIES-1093 (=TAC95)	35	23	25	38	44	41	25	+	+	-	B
**68**	NIES-1213 (=TAC355)	6	49	47	20	45	43	6	+	+	-	B
**69**	NIES-1214 (=TAC356)	20	50	26	20	8	44	6	+	+	-	B
**70**	NIES-1215 (=TAC357)	22	32	48	20	8	45	6	+	+	-	B
**73**	NIES-1126 (=TAC150)	6	51	51	40	46	47	44	+	+	-	B
**77**	NIES-91	20	7	55	6	50	6	6	-	-	-	B
**78**	NIES-1230 (=TAC374)	40	55	56	44	8	39	6	+	+	-	B
**83**	KS1	20	57	60	20	52	45	6	+	+	-	B
**86**	NIES-2472 (=Ks05TA51)	35	59	62	47	54	52	25	+	+	-	B
**88**	Ki05TA02	14	27	63	48	55	53	25	+	+	-	B
**89**	NIES-2473 (=Ki05TA07)	6	60	64	48	55	53	25	+	+	-	B
**91**	NIES-2470 (=Ks05IS11)	35	59	62	49	54	54	6	+	+	-	B
**102**	NIES-2471 (=Ks05IS19)	35	59	62	47	54	23	25	+	+	-	B
**103**	NIES-2624 (=Bi07BH01)	20	62	71	36	33	58	6	-	-	-	B
**107**	SI-2	26	32	73	20	61	6	25	-	-	-	B
**132**	NIES-2552 (=Kw05TA04)	22	11	26	1	33	39	21	-	-	-	B
**133**	NIES-2594 (=Ki05TA03)	20	33	26	63	33	63	6	-	-	-	B
**134**	NIES-2550 (=Ki05TA05)	20	33	81	64	33	64	6	-	-	-	B
**136**	NIES-2494 (=Ki05TA11)	20	33	83	63	36	66	6	-	-	-	B
**137**	NIES-2546 (=Ks05IS01)	20	84	60	20	52	45	6	+	+	-	B
**139**	Ks05IS05	22	85	84	19	33	67	21	-	-	-	B
**141**	NIES-2617 (=Ks05IS12)	35	59	86	65	54	54	25	+	+	-	B
**142**	NIES-2548 (=Ks05IS14)	20	59	62	47	54	52	25	+	+	-	B

**143**	NIES-2618 (=Ks05IS17)	26	32	33	25	32	6	6	-	-	-	B
**148**	Ki05YA04	20	88	89	6	74	71	6	-	-	-	B
**155**	Ii05FU01	20	49	94	64	33	75	6	-	NA	-	B
**159**	NIES-2560 (=Ii05Yo01)	20	95	96	72	74	76	24	-	-	-	B
**160**	Ii05Yo02	6	7	55	6	50	71	6	-	-	-	B
**190**	NIES-1177	14	23	112	88	43	90	44	+	+	NA	B
**192**	NIES-1181	6	51	51	40	46	91	6	+	+	NA	B
**193**	NIES-1183	20	107	94	64	33	64	6	-	-	NA	B
**195**	NIES-2602 (=Ii07Yo01)	20	108	113	6	96	29	6	-	-	-	B
**196**	Ii07Yo02	14	109	64	48	55	53	25	+	+	-	B
**197**	NIES-2088 (=Is07Yo01)	63	110	6	6	97	29	6	-	-	-	B
**218**	Ks08TS08	35	59	62	49	54	96	25	-	-	-	B
**236**	Ai08Si01	20	127	132	75	33	24	6	-	-	-	B
**239**	PCC 7005[g]	71	130	135	19	113	103	93	-	-	-	B
**240**	PCC 9432[g]	71	130	136	100	114	104	94	-	-	-	B
**246**	PCC 9808[g]	6	134	47	20	119	109	6	+	-	-	B
**249**	TAIHU98[g]	20	108	89	6	121	110	100	-	-	-	B
**15**	NIES-109	12	13	12	11	13	13	11	-	-	-	C
**17**	NIES-604	12	14	14	11	15	13	12	-	-	-	C
**50**	NIES-1170 (=TAC198)	12	14	37	32	38	13	34	-	-	-	C
**67**	NIES-1104 (=TAC124)	12	48	46	11	15	42	41	-	-	-	C
**199**	NIES-2603 (=Sw07Hb06)	12	112	115	11	99	13	34	-	-	-	C
**201**	NIES-2623 (=Hw07SP01)	12	14	116	32	99	13	80	-	-	-	C
**8**	NIES-100	7	8	7	7	7	7	7	-	-	-	D
**24**	NIES-1052 (=TAC15)	7	8	20	7	21	16	18	-	-	-	D
**25**	NIES-1059 (=TAC19)	7	8	20	7	21	17	18	-	-	-	D
**27**	NIES-1067 (=TAC65)	7	8	22	7	22	16	19	-	-	-	D
**39**	NIES-1094 (=TAC96)	7	8	30	7	29	17	26	-	-	-	D
**53**	NIES-1252 (=TAC396)	7	39	7	7	7	7	7	-	-	-	D
**59**	NIES-901	7	8	41	7	29	38	18	-	-	-	D
**64**	NIES-1076 (=TAC75)	7	8	22	7	22	17	40	-	-	-	D
**71**	NIES-1232 (=TAC376)	7	8	49	7	29	17	42	-	-	-	D

**79**	NIES-1239 (=TAC383)	7	8	57	7	22	17	26	-	-	-	D
**144**	NIES-2553 (=Ks05YA03)	7	8	49	7	7	69	18	-	-	-	D
**162**	NIES-2619 (=Sn05Hb06)	7	8	98	7	22	17	18	-	-	-	D
**1**	NIES-44[g]	1	1	1	1	1	1	1	-	-	-	E
**12**	NIES-105	11	11	10	1	11	10	1	-	-	NA	E
**14**	NIES-108	11	12	11	1	12	12	1	-	-	-	E
**16**	NIES-112	11	12	13	1	14	1	1	-	-	-	E
**54**	NIES-904	11	40	13	1	39	1	1	-	-	-	E
**58**	NIES-1144 (=TAC171)	34	1	11	1	39	1	1	-	-	-	E
**105**	Kw4	1	1	11	1	1	1	1	-	-	-	E
**138**	NIES-2547 (=Ks05IS04)	11	11	10	1	69	1	1	-	-	-	E
**150**	NIES-2496 (=Kw05YA04)	1	90	91	1	39	1	1	-	-	-	E
**152**	NIES-2555 (=Tw05AK02)	11	92	92	43	14	1	1	-	-	-	E
**156**	NIES-2558 (=Iw05FU01)	1	1	11	70	14	1	1	-	-	-	E
**157**	NIES-2559 (=Iw05FU08)	11	90	10	1	14	1	1	-	-	-	E
**191**	NIES-1179	11	11	10	1	14	1	1	-	-	NA	E
**46**	NIES-1128 (=TAC152)	28	35	25	29	34	31	32	-	-	-	F
**151**	NIES-2554 (=Tw05AK01)	52	91	25	29	76	73	67	-	-	-	F
**153**	NIES=2556 (=Tw05AK10)	53	93	93	29	77	74	67	-	-	-	F
**154**	NIES-2557 (=Tw05AK11)	53	94	25	29	78	31	67	-	-	-	F
**161**	Iw05Yo08	28	91	97	73	80	74	68	-	-	-	F
**235**	NIES-2613 (=Aw08Fu02)	69	126	25	73	76	74	68	-	-	-	F
**30**	NIES-1100 (=TAC114)	19	22	24	18	24	19	22	-	-	-	G
**109**	NIES-2480 (=Ks05TA1)	19	64	74	51	62	60	59	-	-	-	G
**110**	NIES-2481 (=Ks05TA2)[g]	19	65	75	52	62	61	60	-	-	-	G
**111**	NIES-2482 (=Ks05TA3)	45	66	75	53	63	61	59	-	-	-	G
**112**	NIES-2483 (=Ks05TA4)	19	64	76	51	62	22	59	-	-	-	G
**113**	NIES-2484 (=Ks05TA5)	45	67	75	54	63	60	59	-	-	-	G
**114**	NIES-2485 (=Ks05TA6)	19	68	75	55	63	62	59	-	-	-	G
**115**	Ks05TA11	45	69	75	52	64	62	59	-	-	-	G
**116**	NIES-2486 (=Ks05TA21)	45	69	75	52	65	62	59	-	-	-	G
**117**	NIES-2593 (=Ks05TA53)	45	66	75	52	63	62	59	-	-	-	G
**118**	NIES-2487 (=Ks05TA23)	19	70	77	55	66	62	59	-	-	-	G

**119**	Kv05TA01	45	71	75	53	63	61	59	-	-	-	G
**120**	NIES-2549 (=Ks05TA38)[g]	19	65	77	52	62	61	60	-	-	-	G
**121**	NIES-2592 (=Ks05TA28)	45	72	75	56	64	62	59	-	-	-	G
**122**	NIES-2089 (=Ks07TS11)	45	73	75	52	65	60	59	-	-	-	G
**123**	NIES-2488 (=Ks05TA32)	45	74	75	57	63	61	59	-	-	-	G
**124**	Ks05TA33	45	75	75	57	63	61	59	-	-	-	G
**125**	NIES-2489 (=Ks05TA35)	45	76	75	58	63	62	59	-	-	-	G
**126**	NIES-2490 (=Ks05TA37)	45	77	78	59	63	60	59	-	-	-	G
**127**	NIES-2620 (=Ks07TS13)	45	78	75	52	63	62	59	-	-	-	G
**128**	Ks05TA46	45	79	75	57	63	61	59	-	-	-	G
**129**	NIES-2491 (=Ks05TA52)	19	80	79	60	63	60	59	-	-	-	G
**169**	NIES-2596 (=Ks07TS20)	19	101	77	77	85	62	59	-	-	-	G
**170**	NIES-2597 (=Ks07TS27)	19	70	77	55	86	62	59	-	-	-	G
**171**	NIES-2090 (=Ks07TS29)	19	65	77	52	62	62	60	-	-	-	G
**172**	Ks07TS43	45	64	76	52	65	62	59	-	-	NA	G
**173**	NIES-2091 (=Ks07TS48)	45	102	74	52	66	60	59	-	-	-	G
**175**	Ks07TS93	19	79	77	78	86	83	59	-	-	-	G
**176**	Ks07TS99	19	68	105	79	66	62	59	-	-	-	G
**177**	Ks07TS102	19	102	74	80	66	60	59	-	-	-	G
**178**	Ks07TS105	19	66	75	52	65	62	59	-	-	-	G
**179**	NIES-2599 (=Ks07TS123)	45	102	75	52	65	62	59	-	-	-	G
**180**	NIES-2622 (=Ks07TS127)	45	76	106	52	64	62	59	-	-	-	G
**182**	Ks07TS139	19	103	107	81	88	85	28	-	-	-	G
**212**	Ka08TS01	19	117	123	52	66	62	59	-	-	-	G
**213**	Ka08TS02	19	101	124	55	85	22	59	-	-	-	G
**215**	Ks08TS03	45	69	125	52	65	62	59	-	-	-	G
**217**	NIES-2607 (=Ks08TS06)	19	68	105	94	66	62	59	-	-	-	G
**219**	NIES-2626 (=Ks08YA11)	45	67	127	66	63	97	59	-	-	-	G
**221**	NIES-2608 (=Ks08YA15)	19	70	128	55	106	62	59	-	-	-	G
**222**	Ks08YA16	19	74	74	52	65	62	59	-	-	-	G
**223**	Ks08YA22	19	70	75	55	106	62	59	-	-	-	G
**224**	Ks08YA25	19	119	123	52	66	62	59	-	-	-	G
**227**	NIES-2628 (=As08Gb04)	19	121	123	52	65	60	59	-	-	-	G

**2**	NIES-87	2	2	2	2	2	2	2	-	-	-	H
**38**	NIES-1098 (=TAC110)	2	2	2	2	28	2	2	-	-	-	H
**104**	KI-3	2	63	72	2	60	59	2	-	-	-	H
**146**	Ki05YA02	49	87	88	68	72	70	65	-	-	-	H
**147**	Ki05YA03	50	63	2	2	73	59	2	-	-	-	H
**164**	NIES-2595 (=Ss05Mb06)	49	96	99	68	81	78	65	-	-	-	H
**166**	Thvi8	56	98	101	2	60	80	70	-	-	-	H
**174**	Ks07TS52c	59	96	104	68	72	78	65	-	NA	-	H
**210**	Ki08TS01	64	115	121	2	103	95	85	-	NA	-	H
**226**	As08Gb02	64	2	2	2	28	2	89	-	-	-	H
**228**	NIES-2612 (=As08Gb10)	49	122	121	37	107	78	90	-	-	-	H
**140**	Ks05IS06	47	86	85	66	70	68	63	-	-	-	I
**187**	NIES-2621 (=Kw07TS101)	47	86	85	86	93	68	76	-	-	-	I
**220**	Ks08YA14	47	86	85	94	105	68	88	-	-	-	I
**74**	NIES-1132 (=TAC156)	37	52	52	41	47	48	45	-	-	-	J
**130**	NIES-2492 (=Ks05TA56)	46	81	80	61	67	16	61	-	-	-	J
**181**	NIES-2600 (=Ks07TS137)	46	81	80	61	87	84	61	-	-	-	J
**184**	NIES-2601 (=Ks07TS159)	61	81	109	83	90	87	74	-	-	-	J
**208**	SKs08Zn11	46	81	120	61	90	84	84	-	-	NA	J
**232**	Aa08Fu02	67	81	52	61	90	48	61	-	-	-	J
**244**	PCC 9806[g]	67	81	139	102	90	107	97	-	NA	-	J
**23**	T20-3	16	19	19	15	20	15	17	+	+	NA	X
**57**	NIES-1143 (=TAC170)	33	43	40	34	41	37	39	+	+	-	X
**95**	Tn05AK03	33	43	67	20	41	57	53	+	+	-	X
**205**	Rs08SH03	33	43	67	34	41	57	39	+	+	NA	X
**206**	NIES-2605 (=Rs08NA05)	33	113	118	90	101	93	82	+	+	NA	X
**241**	PCC 9443[g]	33	131	137	101	115	105	95	+	NA	-	X
**245**	PCC 9807[g]	73	133	140	103	118	108	98	+	+	-	X
**11**	NIES-104	10	10	9	9	10	9	9	-	-	-	NA
**34**	NIES-1050 (=TAC4)	21	26	27	21	26	22	23	-	-	-	NA
**40**	NIES-1101 (=TAC115)	24	30	31	23	30	25	27	-	-	-	NA
**41**	NIES-1106 (=TAC126)	25	31	32	24	31	26	28	-	-	-	NA

**47**	NIES-1211[g] (=TAC352)	29	36	35	30	35	32	33	-	-	+	NA
**56**	NIES-1142 (=TAC169)	32	42	39	33	40	36	38	-	-	-	NA
**75**	NIES-1130 (=TAC154)	38	53	53	42	48	49	46	-	-	-	NA
**131**	NIES-2551 (=Kw05TA03)	10	82	9	62	68	9	9	-	-	-	NA
**135**	NIES-1176	14	83	82	65	25	65	62	-	-	-	NA
**145**	Ki05YA01	48	36	87	67	71	32	64	-	-	-	NA
**149**	NIES-2495 (=Kw05YA03)	51	89	90	69	75	72	66	-	-	-	NA
**158**	Ia05Yo06	54	36	95	71	79	32	3	-	NA	-	NA
**165**	Thvi7	55	97	100	74	82	79	69	-	-	-	NA
**167**	CTS3-5	57	99	102	75	83	81	71	-	-	-	NA
**168**	CTS3-8	58	100	103	76	84	82	72	-	-	-	NA
**183**	Ks07TS141	60	104	108	82	89	86	73	-	-	-	NA
**186**	Kn07TS121	62	105	110	85	92	88	75	-	-	-	NA
**189**	NIES-1175	10	106	111	9	95	9	77	-	-	NA	NA
**200**	Hs07SP05	48	36	116	67	100	32	64	-	-	-	NA
**207**	Rw08NA01	24	30	119	23	102	94	83	-	NA	NA	NA
**209**	NIES-2625 (=SKw08Ya04)	10	114	111	91	68	9	9	-	-	NA	NA
**211**	Ki08TS02	65	116	122	92	104	67	86	-	-	-	NA
**216**	NIES-2606 (=Ks08TS05)	60	118	126	82	89	62	87	-	-	-	NA
**225**	As08Gb01	60	120	126	95	89	86	87	-	-	-	NA
**231**	NIES-2629 (=As08Hj03)	60	125	130	96	89	62	87	-	-	-	NA
**233**	Ai08Fu01	68	100	131	97	110	22	72	-	-	-	NA
**237**	PCC 7806[g]	20	128	133	98	111	101	91	+	+	+	NA
**238**	Sj[g]	70	129	134	99	112	102	92	+	+	+	NA
**242**	PCC 9701[g]	72	99	138	75	116	95	96	-	NA	-	NA
**248**	T1-4[g]	74	36	142	67	120	32	99	-	NA	-	NA

[a] Sequence type. ST1-237 data are according to the previous study (Tanabe et al., 2011).

[b] Each allele number indicates a different sequence.

[c] PCR detection of *mcyG* (Tanabe et al., 2009) or genomic evidence of *mcy* presence if available.

[d] Microcystins were detected following the published protocol (Tanabe et al., 2009b). Published data are based on Tanabe et al., (2009b) and Humbert et al., (2013). NA, not analyzed or not available.

[e] PCR detection of three sucrose genes or genomic evidence of the genes (Sj and PCC 7806). NA, not analyzed.

[f] Group assignment based on MLST phylogeny (Fig. 2) with reference to a preivous study (Tanabe et al., 2011). NA, strains assigned to neither group by phylogenetic analyses.

[g] Whole or draft genome sequence is available.

[h] Imcomplete *mcy* gene cluster (Humbert et al., 2013).
