# Supplementary material for: Adaptation of the Freshwater Bloom-Forming Cyanobacterium Microcystis aeruginosa to Brackish Water Is Driven by Recent Horizontal Transfer of Sucrose Genes
Source: Front Microbiol. 2018 Jun 5;9:1150. doi: 10.3389/fmicb.2018.01150 (PMC5996124; doi:10.3389/fmicb.2018.01150)
Supplement: Supplementary file 3 [file Table_3.PDF]

Supplementary Table S3. RT-qPCR primers used in this study.

Locus	Gene product	Primer	Amplicon size (bp)	Sequence (5'-3')	Reference
sppA	Sucrose-phosphate phosphatase	sppA_F	121	ggCgTTTCCgACgATTACCC	This study
		sppA_R		ggCCACCTATCTgCgTCAgT	This study
spsA	Sucrose-phosphate synthase	spsA_F	135	CCATAATCgCACgggTCTgC	This study
		spsA_R		AAACgggACTCgACCCACTC	This study
susA	Sucrose synthase	susA_F	133	AgTCTTTggTCgCAgCgAgT	This study
		susA_R		CCCgCAgCTTggAACAACAA	This study
mcyA	Microcystin synthetase A	mcyA_F	133	AggAgCCggCgAACATAACA	This study
		mcyA_R		TgggCCgAgTCgAAAgAgTC	This study
mcyE	Microcystin synthetase E	mcyE_F	117	gTTggCTggAATAgCAgTTACACg	This study
		mcyE_R		ACCCggTTCCACAACCTATTTCC	This study
rnpB	RNA component of RNAse P	rnpB_Fw[a]	104	ggggTAAgggTgCAAAggT	Makower et al, (2015)
(reference)		rnpB_Rv[a]		AgACCAACCTTTgTCCCTCC	Makower et al, (2015)

[a] The sequences targeted by the primer are perfectly conserved among PCC 7806, Sj, and NIES-1211.
