# Supplementary material for: Adaptation of the Freshwater Bloom-Forming Cyanobacterium Microcystis aeruginosa to Brackish Water Is Driven by Recent Horizontal Transfer of Sucrose Genes
Source: Front Microbiol. 2018 Jun 5;9:1150. doi: 10.3389/fmicb.2018.01150 (PMC5996124; doi:10.3389/fmicb.2018.01150)
Supplement: Supplementary file 4 [file Table_4.PDF]

Supplementary Table S4. PCR primers for sucrose synthesis gene detection and genotyping.

Locus	Gene product	Primer	Amplicon size (bp)	Annealing temperature	Sequence (5'-3')
spsA	Sucrose-phosphate synthase	spsA_1F	430	67 °C	CCAAAgTCgTTCCCAgTgTT
		spsA_1R			ATTgACCAAggTTTCgATgC
		spsA_3F	498	60 °C	ATCATTCCCTTgCTgCAATC
		spsA_3R			CggCgTAgTAgCTgggTAAg
susA	Sucrose synthase	susA_1F	486	60 °C	ACCgAATCAAAAACCTgACg
		susA_1R			CAgAAATTTCCCgCCAgTAA
		susA_2F	493	60 °C	AACggCAACTATCCCAACAg
		susA_2R			ATgCAggCATTATCCgTACC
sppA	Sucrose-phosphate phosphatase	sppA_1F	462	60 °C	ggTgAAgTTgATCCCCAATg
		sppA_1R			gggggATTTTgCCAgATAAT
