# Supplementary material for: Adaptation of the Freshwater Bloom-Forming Cyanobacterium Microcystis aeruginosa to Brackish Water Is Driven by Recent Horizontal Transfer of Sucrose Genes
Source: Front Microbiol. 2018 Jun 5;9:1150. doi: 10.3389/fmicb.2018.01150 (PMC5996124; doi:10.3389/fmicb.2018.01150)
Supplement: Supplementary file 5 [file Table_5.PDF]

Supplementary Table S5. List of putative genes for salt tolerance.[a]

Gene	Gene product	Sj	NIES-1211	PCC 7806	NIES-843
bicA (sll0834)	Sodium-dependent bicarbonate transporter	MSj_00607	MTo_01952	IPF_4911	-
sbtA (slr1512)	Sodium-dependent bicarbonate transporter	-	MTo_01953	-	MAE_62090
sbtB	Sodium-dependent bicarbonate transporter	-	MTo_01954	-	MAE_62100
nhaS1 (slr1727)	Sodium/proton antiporter	MSj_03083	MTo_01887	IPF_4370	MAE_36160
-	Sodium/proton antiporter	MSj_01433	MTo_01057	IPF_1669	MAE_38000
nhaS2 (sll0273)	Sodium/proton antiporter	MSj_03540	-	IPF_5459	-
nhaS3 (sll0689)	Sodium/proton antiporter	MSj_00606	MTo_01955	IPF_4912	MAE_62110
nhaS3	Sodium/proton antiporter	MSj_01662	MTo_02487	BH695_1123	MAE_55560
nhaS4 (slr1595)	Sodium/proton antiporter	-	-	-	-
nhaS5 (slr0415)	Sodium/proton antiporter	-	-	-	-
nhaS6 (sll0556)	Sodium/proton antiporter	MSj_00948	MTo_02757	IPF_4609, 7963	MAE_60970
slr0753	Sodium/proton antiporter (putative)	MSj_02250	MTo_01183	IPF_4390	MAE_59110
sll1087	Sodium/solute symporter	MSj_03623	MTo_04210	IPF_871 -641	MAE_10360
mrp operon	Multisubunit sodium/proton antiporter	MSj_01845 -01851	MTo_02941 -02948	IPF_634 -641	MAE_23720 -23770
gltS (slr1145)	Sodium-dependent glutamate trasnporter	MSj_01313	MTo_03060	IPF_5465	MAE_13630

sll1428	Putative sodium-dependent transporter	MSj_00039	MTo_01992	IPF_5556	MAE_16140
slr0625	Sodium/glutamate symporter	-	-	-	-
slr0681	Sodium/calcium exchanger	-	-	-	-
gtrABC (sll1102-1104)	Sodium-dependent glutamate trasnport	-	-	-	-
sac1 (sll0640)	Sodium/sulfate symporter	MSj_02182	MTo_02029	IPF_2980-2981	MAE_12960
sll0993 (*kchX*)	Potassium channel	MSj_02014	MTo_04250	IPF_4613	MAE_15730
kdpA (slr1728)	Potassium-transporting P-type ATPase A chain	MSj_03290	MTo_03821	IPF_1236	MAE_59800
kdpB (slr1729)	Potassium-transporting P-type ATPase B chain	MSj_03289	MTo_03822	IPF_1235	MAE_59820
kdpC (slr1730)	Potassium-transporting P-type ATPase C chain	MSj_03286	MTo_03711	IPF_1229	MAE_59870
kdpD (slr1731)	Potassium-transporting P-type ATPase D chain	MSj_01124	MTo_04023	IPF_5006	MAE_53250
slr1509 *(ktrB/ntpJ)*	Membrane subunit of a Ktr-like ion transport system	MSj_02839	MTo_00368	IPF_4143	MAE_18660
sll0261	Potassium channel	MSj_01316	MTo_01528	IPF_2272	MAE_35050
trkA (slr0773)	Trk system potassium uptake protein	MSj_03541	-	IPF_5457	-
trkA (sll0493)	Trk system potassium uptake protein	MSj_02838	MTo_00369	IPF_4144	MAE_18650
slr5078	Potassium channel protein	-	-	-	-
slr0801	Putative flavoprotein involved in potassium transport	-	-	-	-
sll0536	Potassium channel protein	MSj_03115	MTo_02872	IPF_1361	MAE_30200
-	Potassium channel	MSj_01497	MTo_02239	IPF_4452	MAE_06710

eriC (sll1864)	Chloride channel protein	MSj_00165	MTo_01550	IPF_3699	MAE_38710
sll0855	Chloride channel protein	-	-	-	-
sll0103	Chloride-activated chloride channel homologue	MSj_01522	MTo_03009	IPF_3014	MAE_50360
slr7060	Chloride-activated chloride channel homologue	-	-	-	-
spsA (sll0045)	Sucrose phosphate synthase	MSj_02703	MTo_03975	IPF_1564	-
susA	Sucrose synthase	MSj_02704	MTo_03976	IPF_1565	-
sppA (slr0953)	Sucrose phosphate phosphatase	MSj_02705	MTo_03977	IPF_1566	-
treY	Maltooligosyl trehalose synthase	MSj_01016[b]	MTo_00063[b]	IPF_5153[b]	MAE_17300[b]
treZ	Maltooligosyl trehalohydrolase	-	-	-	-
treS	Trehalose synthase/amylase	-	-	-	-
otsA	Trehalose-6-phosphate synthase	-	-	-	-
otsB	Trehalose-phosphate phosphatase	-	-	-	-
ggpS (sll1566)	Glucosylglycerolphosphate synthase	-	-	-	-
ggpP (slr0746)	Glucosylglycerolphosphate synthase	-	-	-	-
ggtA (slr0747)	Glucosylglycerol transport system ATP-binding protein	-	-	-	-
ggtB (slr0529)	lucosylglycerol transport system substrate-binding protei	-	-	-	-
ggtC (slr0530)	Glucosylglycerol transport system permease protein	-	-	-	-
ggtD (slr0531)	Glucosylglycerol transport system permease protein	-	-	-	-

mscS (slr0765)	Small-conductance mechanosensitive channel	MSj_02984	MTo_02574	IPF_1474	MAE_53900
mscS (slr0639)	Small-conductance mechanosensitive channel	MSj_00745	MTo_01710	IPF_829	MAE_44000
mscL (slr0875)	Large-conductance mechanosensitive channel	-	-	-	-
aqpZ (slr2057)	Water channel protein	-	-	-	MAE_09800
aqpZ	Water channel protein	-	-	IPF_3590	-
actM	Actin	-	-	IPF_5084	-
pfnM	Profilin	-	-	IPF_5083	-

[a] The list is based on the published studies (Klähn and Hagemann, 2011; Sandrini et al., 2015) with several additional genes.
[b] *treY* homolog is detected. However, *treZ* is needed for the synthesis of trehalose (Klähn and Hagemann, 2011).
