# Supplementary material for: Adaptation of the Freshwater Bloom-Forming Cyanobacterium Microcystis aeruginosa to Brackish Water Is Driven by Recent Horizontal Transfer of Sucrose Genes
Source: Front Microbiol. 2018 Jun 5;9:1150. doi: 10.3389/fmicb.2018.01150 (PMC5996124; doi:10.3389/fmicb.2018.01150)
Supplement: Supplementary file 6 [file Table_6.PDF]

Supplementary Table S6. MLST and core genome genes included in Figure 4.

Sj	NIES-1211	PCC 7807	Category[a]	Gene and/or products
MSj_03772	MTo_00952	IPF_2605	MLST	*ftsZ* , cell division protein FtsZ
MSj_01163	MTo_02868	IPF_3887	MLST	*glnA* , glutamine synthetase
MSj_01831	MTo_00554	IPF_2806	MLST	*gltX* , glutamyl-tRNA synthetase
MSj_00926	MTo_00680	IPF_2537	MLST	*gyrB* , DNA gyrase subunit B
MSj_02529	MTo_02100	IPF_942	MLST	*pgi* , glucose-6-phosphate isomerase
MSj_00037	MTo_01994	IPF_5554	MLST	*recA* , recombination protein RecA
MSj_00302	MTo_04129	IPF_5206	MLST	*tpi* , triosephosphate isomerase
MSj_02706	MTo_03978	IPF_1567		*leuS* , leucyl-tRNA synthetase
MSj_02419	MTo_02024	IPF_2513	PhyloSift	Ribosomal protein S2
MSj_03426	MTo_01726	IPF_743	PhyloSift	*rplA* , ribosomal protein L1
MSj_00401	MTo_03773	IPF_4570	PhyloSift	Translation initiation factor IF2
MSj_03285	MTo_01226	IPF_4642	PhyloSift	Metalloendopeptidase
MSj_00247	MTo_01299	IPF_3296	PhyloSift	*rplB, r* ibosomal protein L2
MSj_03246	MTo_02441	IPF_3195	PhyloSift	Phenylalanyl-tRNA synthetase alpha subunit
MSj_03054	MTo_01474	IPF_1537	PhyloSift	Phenylalanyl-tRNA synthetase beta subunit
MSj_00250	MTo_01302	IPF_3292	PhyloSift	*rpsC* , ribosomal protein S3
MSj_02047	MTo_00310	IPF_2742	PhyloSift	tRNA pseudouridine synthase B
MSj_01393	MTo_03785	IPF_4407	PhyloSift	Porphobilinogen deaminase
MSj_00315	MTo_02371	IPF_5476	OrthoVenn	Nitrate reductase
MSj_01512	MTo_02253	IPF_4469	OrthoVenn	Methionine aminopeptidase
MSj_02343	MTo_03219	IPF_5731	OrthoVenn	Uncharacterized aminotransferase
MSj_02531	MTo_02098	IPF_1698	OrthoVenn	Histidine biosynthesis trifunctional protein
MSj_03952	MTo_02296	IPF_1812	OrthoVenn	ABC transporter ATP-binding protein/permease VM
MSj_00411	MTo_00768	IPF_2456	OrthoVenn	Dihydroorotase
MSj_01341	MTo_03185	IPF_156	OrthoVenn	mRNA export factor elf1
MSj_02698	MTo_02117	IPF_2055	OrthoVenn	Heat shock protein SSA2
MSj_00036	MTo_01995	IPF_7408	OrthoVenn	Putative aryl-alcohol dehydrogenase
MSj_02722	MTo_02212	IPF_3552	OrthoVenn	ABC transporter ATP-binding protein/permease VM
MSj_02437	MTo_00846	IPF_3625	OrthoVenn	Bifunctional purine biosynthetic protein

MSj_01632	MTo_03592	IPF_2177	OrthoVenn	Elongation factor 3
MSj_03092	MTo_02325	IPF_598	OrthoVenn	Asparagine-tRNA ligase
MSj_00326	MTo_02618	IPF_3536	OrthoVenn	Heat shock protein STI1
MSj_01612	MTo_00009	IPF_3052	OrthoVenn	Glutamate-5-kinase
MSj_01526	MTo_03023	IPF_3008	OrthoVenn	Gamma-glutamyl phosphate reductase
MSj_01832	MTo_00553	IPF_2807	OrthoVenn	ATP-dependent 6-phosphofructokinase subunit beta
MSj_00405	MTo_03777	IPF_4574	OrthoVenn	ATP-dependent zinc metalloprotease FtsH
MSj_03797	MTo_00510	IPF_3084	OrthoVenn	Putative cysteine synthase
MSj_03377	MTo_01231	IPF_4650	OrthoVenn	Putative protein kinase UbiB
MSj_01449	MTo_02419	IPF_457	OrthoVenn	Arginine-tRNA ligase
MSj_03650	MTo_03152	IPF_1080	OrthoVenn	ATP-binding cassette transporter abc3
MSj_03150	MTo_03482	IPF_1548	OrthoVenn	NADH-ubiquinone oxidoreductase chain 5
MSj_01918	MTo_00655	IPF_2711	OrthoVenn	Argininosuccinate synthase
MSj_01941	MTo_03122	IPF_2242	OrthoVenn	Kinesin-like protein cut7
MSj_03217	MTo_00796	IPF_4565	OrthoVenn	Deoxyribodipyrimidine photo-lyase
MSj_02173	MTo_03304	IPF_2990	OrthoVenn	Serine-tRNA ligase
MSj_02660	MTo_01614	IPF_1243	OrthoVenn	Translation factor GUF1
MSj_03820	MTo_00842	IPF_833	OrthoVenn	Proline-tRNA ligase
MSj_01056	MTo_01878	IPF_6652	OrthoVenn	Putative NADPH dehydrogenase
MSj_00543	MTo_01688	IPF_2448	OrthoVenn	Glucose-6-phosphate 1-dehydrogenase
MSj_03327	MTo_03574	IPF_4093	OrthoVenn	Putative aldehyde dehydrogenase-like protein
MSj_01459	MTo_04444	IPF_445	OrthoVenn	Acetylornithine aminotransferase
MSj_01629	MTo_03589	IPF_2174	OrthoVenn	26S protease regulatory subunit 8 homolog
MSj_01166	MTo_01676	IPF_3892	OrthoVenn	NAD kinase
MSj_03358	MTo_03688	IPF_4861	OrthoVenn	Tyrosine-tRNA ligase
MSj_02185	MTo_02032	IPF_2973	OrthoVenn	Bifunctional lycopene cyclase/phytoene synthase
MSj_00188	MTo_01563	IPF_5236	OrthoVenn	Uridine kinase
MSj_02684	MTo_02103	IPF_1726	OrthoVenn	Serine hydroxymethyltransferase
MSj_03473	MTo_02977	IPF_796	OrthoVenn	Heat shock protein
MSj_01207	MTo_03668	IPF_2873	OrthoVenn	Ornithine carbamoyltransferase
MSj_03148	MTo_03480	IPF_1546	OrthoVenn	NADH-ubiquinone oxidoreductase chain 4
MSj_03073	MTo_01897	IPF_5247	OrthoVenn	3-ketodihydrosphingosine reductase gsl-3

MSj_02443	MTo_01818	IPF_83	OrthoVenn	Histidinol-phosphate aminotransferase
MSj_00115	MTo_02591	IPF_3511	OrthoVenn	Elongation factor 3
MSj_03083	MTo_01887	IPF_4370	OrthoVenn	Na(+)/H(+) antiporter NhaS1
MSj_01349	MTo_03177	IPF_148	OrthoVenn	Subtilisin-like protease 10
MSj_01767	MTo_00491	IPF_4263	OrthoVenn	26S protease regulatory subunit 6B homolog
MSj_00472	MTo_03609	IPF_629	OrthoVenn	Glutamyl-tRNA(Gln) amidotransferase subunit B
MSj_01382	MTo_02283	IPF_2228	OrthoVenn	Methionine-tRNA ligase
MSj_01700	MTo_02193	IPF_4153	OrthoVenn	Nicotinate-nucleotide pyrophosphorylase
MSj_02029	MTo_00290	IPF_2760	OrthoVenn	Aminodeoxychorismate synthase
MSj_00718	MTo_01156	IPF_3351	OrthoVenn	Aminomethyltransferase
MSj_00626	MTo_02003	IPF_4924	OrthoVenn	Heat shock 70 kDa protein
MSj_01252	MTo_01639	IPF_4224	OrthoVenn	GTP cyclohydrolase-2
MSj_00345	MTo_03401	IPF_286	OrthoVenn	Pre-mRNA-splicing ATP-dependent RNA helicase
MSj_00916	MTo_01666	IPF_4496	OrthoVenn	Putative truncated L-serine dehydratase
MSj_00185	MTo_01565	IPF_4478	OrthoVenn	Methylthioribose-1-phosphate isomerase
MSj_03764	MTo_01354	IPF_4831	OrthoVenn	L-lactate dehydrogenase B
MSj_03027	MTo_02655	IPF_3570	OrthoVenn	Malate dehydrogenase

[a]Abbreviations: MLST, the MLST loci (Tanabe et al., 2007); PhyloSift, genes selected in PhyloSift (Darling et al., 2014), OrthoVenn, genes randomly chosen from the result of OrthoVenn analysis (Wang et al., 2015).

Reference

Wang, Y, Coleman-Derr, D., Chen, G., and Gu, Y. Q. (2015). OrthoVenn: a web server for genome wide comparison and annotation of orthologous clusters across multiple species. *Nucleic Acids Res.* 43, W78–W84.
