# Supplementary material for: Adaptation of the Freshwater Bloom-Forming Cyanobacterium Microcystis aeruginosa to Brackish Water Is Driven by Recent Horizontal Transfer of Sucrose Genes
Source: Front Microbiol. 2018 Jun 5;9:1150. doi: 10.3389/fmicb.2018.01150 (PMC5996124; doi:10.3389/fmicb.2018.01150)
Supplement: Supplementary file 7 [file Image_1.PDF]

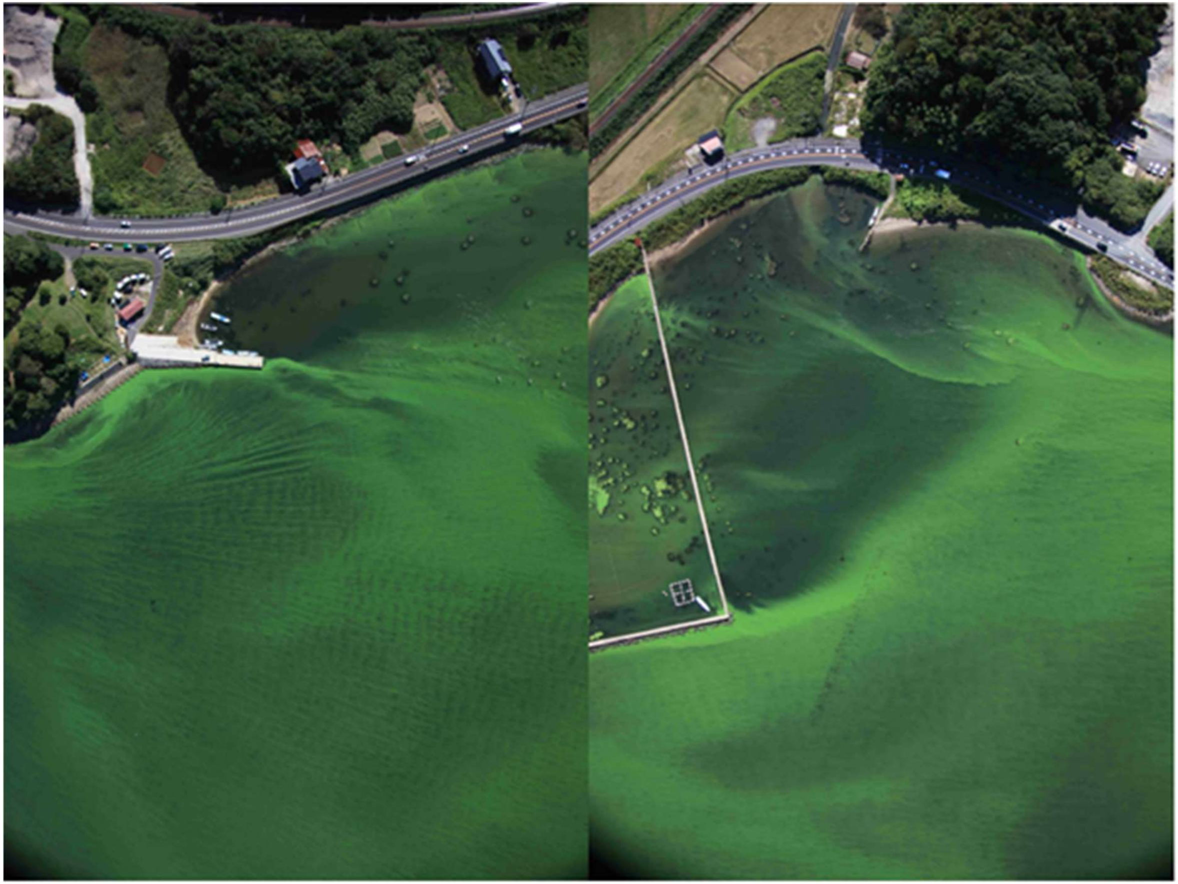

Supplementary Figure S1. Bird's-eye views of *M. aeruginosa* bloom in Lake Shinji. The photo was taken on October 7, 2010**.**
