# Supplementary material for: Adaptation of the Freshwater Bloom-Forming Cyanobacterium Microcystis aeruginosa to Brackish Water Is Driven by Recent Horizontal Transfer of Sucrose Genes
Source: Front Microbiol. 2018 Jun 5;9:1150. doi: 10.3389/fmicb.2018.01150 (PMC5996124; doi:10.3389/fmicb.2018.01150)
Supplement: Supplementary file 8 [file Image_2.PDF]

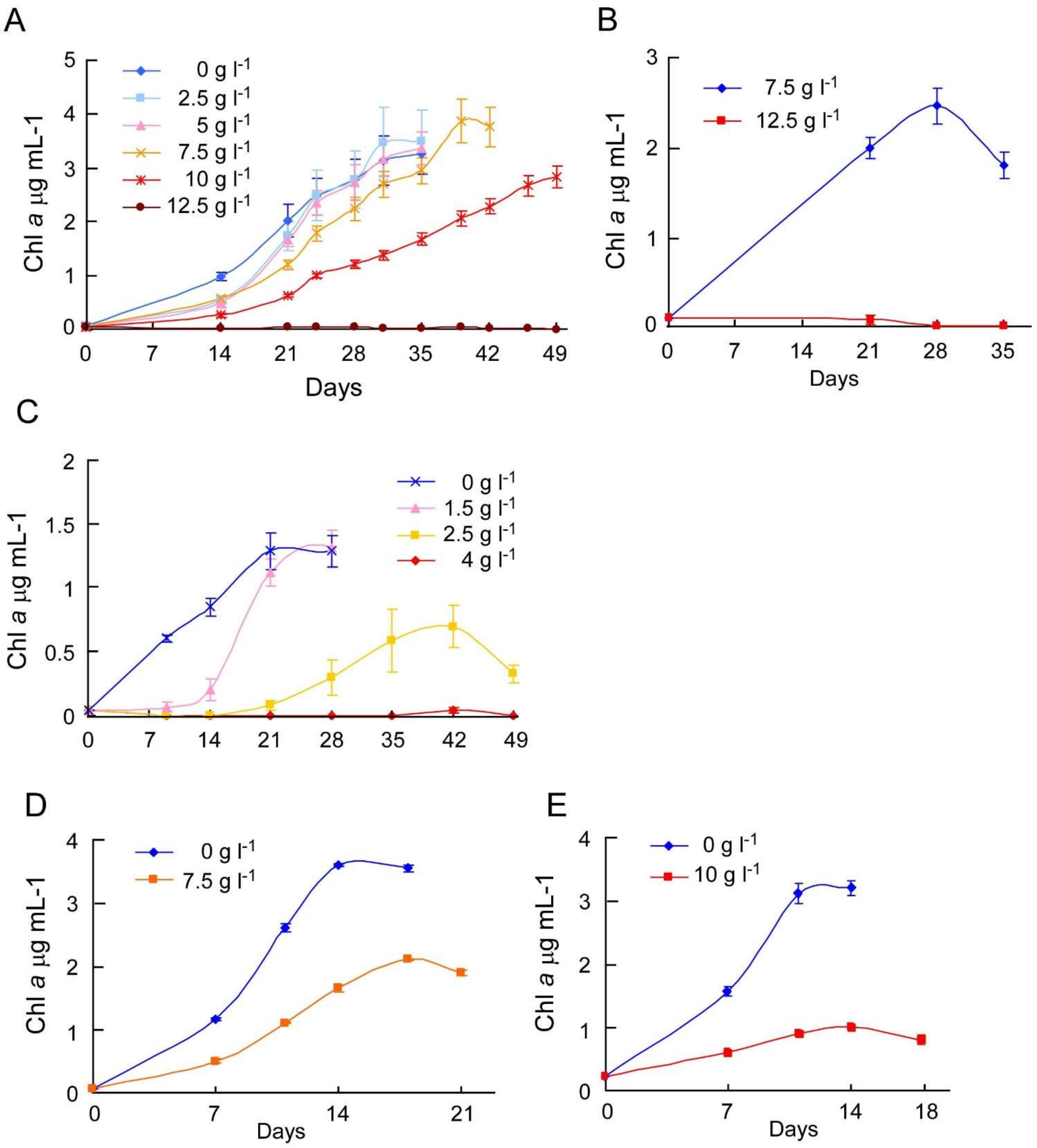

Supplementary Figure S2. Growth curves of *M. aeruginosa* strains. **A**, Growth curves of Sj under different NaCl concentrations. **B**, Growth curves of Sj using a salt-acclimated culture as the seed (precultured in MA with 7.5 g l^{-1} NaCl). **C**, Growth curves of NIES-843 under different NaCl concentrations. **D**, **E**, Growth curves of NIES-1211 under different NaCl concentrations. Bars indicate the standard error of five (**A**) and three (**B**, **C**, **D, E**) biological replicates.
