# Supplementary material for: Adaptation of the Freshwater Bloom-Forming Cyanobacterium Microcystis aeruginosa to Brackish Water Is Driven by Recent Horizontal Transfer of Sucrose Genes
Source: Front Microbiol. 2018 Jun 5;9:1150. doi: 10.3389/fmicb.2018.01150 (PMC5996124; doi:10.3389/fmicb.2018.01150)
Supplement: Supplementary file 9 [file Image_3.PDF]

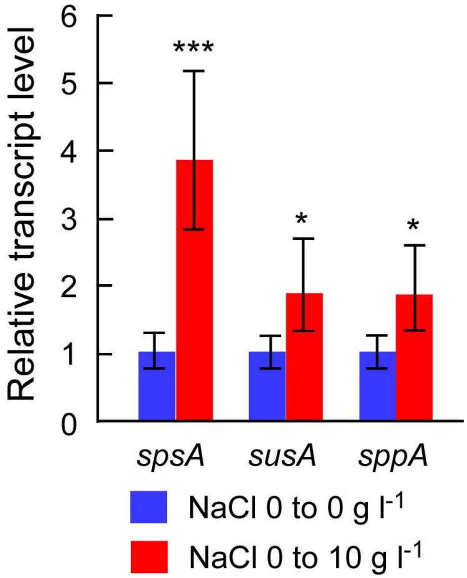

Supplementary Figure S3. Results of RT-qPCR experiments for the sucrose genes of NIES-1211. The expression of *rnpB* was used as a reference (Makower et al., 2015). Bars indicate 95 % confidence intervals, as in Figure 3. Statistical significance by homoscedastic one-tailed t-tests: *, $P < 0.05$; ***, $P < 0.001$.
