# Supplementary material for: Adaptation of the Freshwater Bloom-Forming Cyanobacterium Microcystis aeruginosa to Brackish Water Is Driven by Recent Horizontal Transfer of Sucrose Genes
Source: Front Microbiol. 2018 Jun 5;9:1150. doi: 10.3389/fmicb.2018.01150 (PMC5996124; doi:10.3389/fmicb.2018.01150)
Supplement: Supplementary file 10 [file Image_4.PDF]

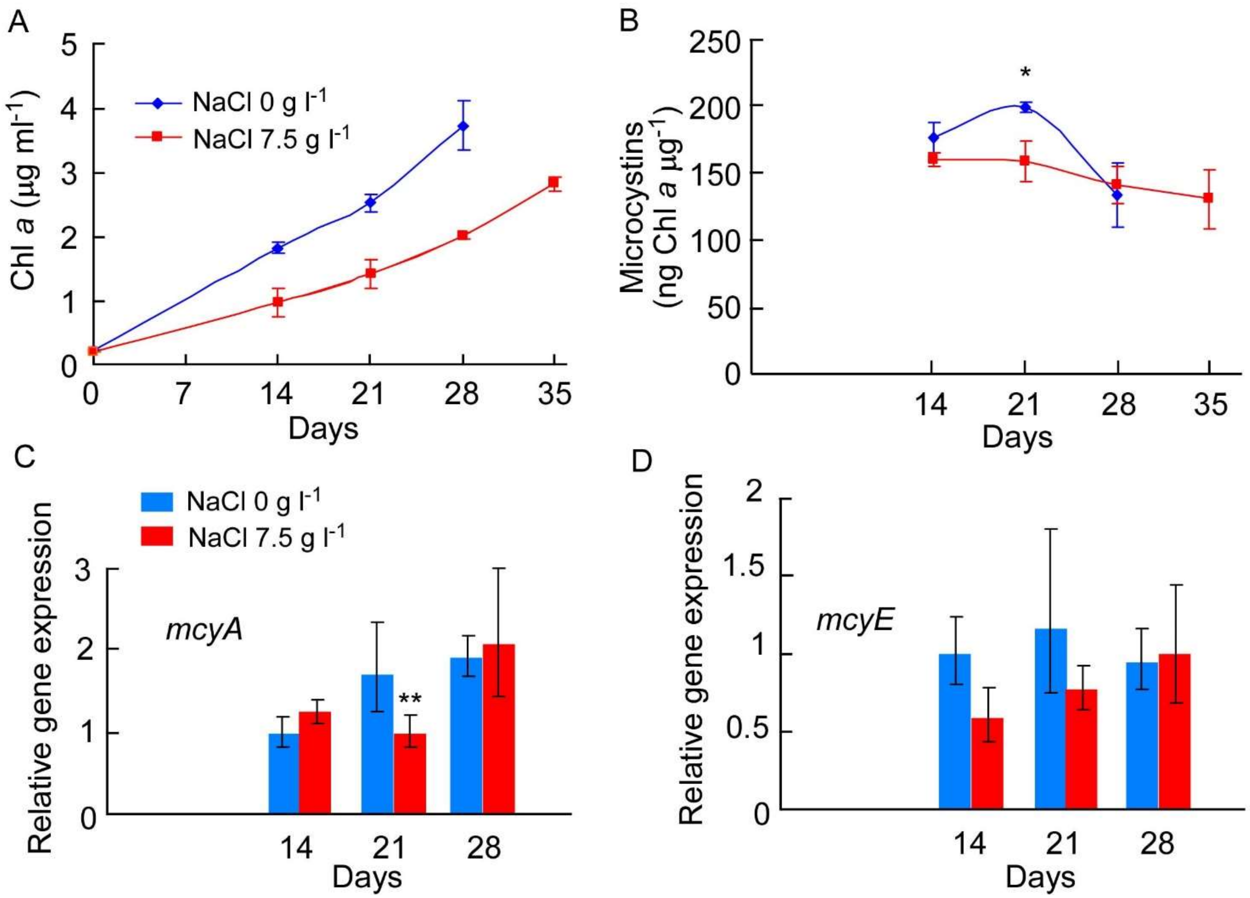

Supplementary Figure S4. Microcystin concentrations of Sj cultures during the growth period. **A**, Growth curves of Sj under different NaCl concentrations. **B**, Microcystin concentrations at the time points of **A**. **C**, **D**, *mcyA* and *mcyE* gene expression levels, respectively, at the time points of **A**. The two *mcy* genes were selected for RT-qPCR analyses because they were shown to be in different transcripts (Kaebernick et al., 2000). Bars indicate the standard errors of three biological replicates (**A**, **B**) and 95 % confidence intervals (**C**, **D**), respectively, as in Figure 3. Statistical significance by homoscedastic one-tailed t-tests: *, $P < 0.05$; **, $P < 0.01$. Results of t-tests for **A** were not shown.

Reference

Kaebernick, M., Neilan, B. A., Börner T., and Dittmann, E. (2000). Light and the transcriptional response of the microcystin biosynthesis gene cluster. *Appl. Environ. Microbiol.* 66, 3387–3392.
