# Supplementary material for: Adaptation of the Freshwater Bloom-Forming Cyanobacterium Microcystis aeruginosa to Brackish Water Is Driven by Recent Horizontal Transfer of Sucrose Genes
Source: Front Microbiol. 2018 Jun 5;9:1150. doi: 10.3389/fmicb.2018.01150 (PMC5996124; doi:10.3389/fmicb.2018.01150)
Supplement: Supplementary file 11 [file Image_5.PDF]

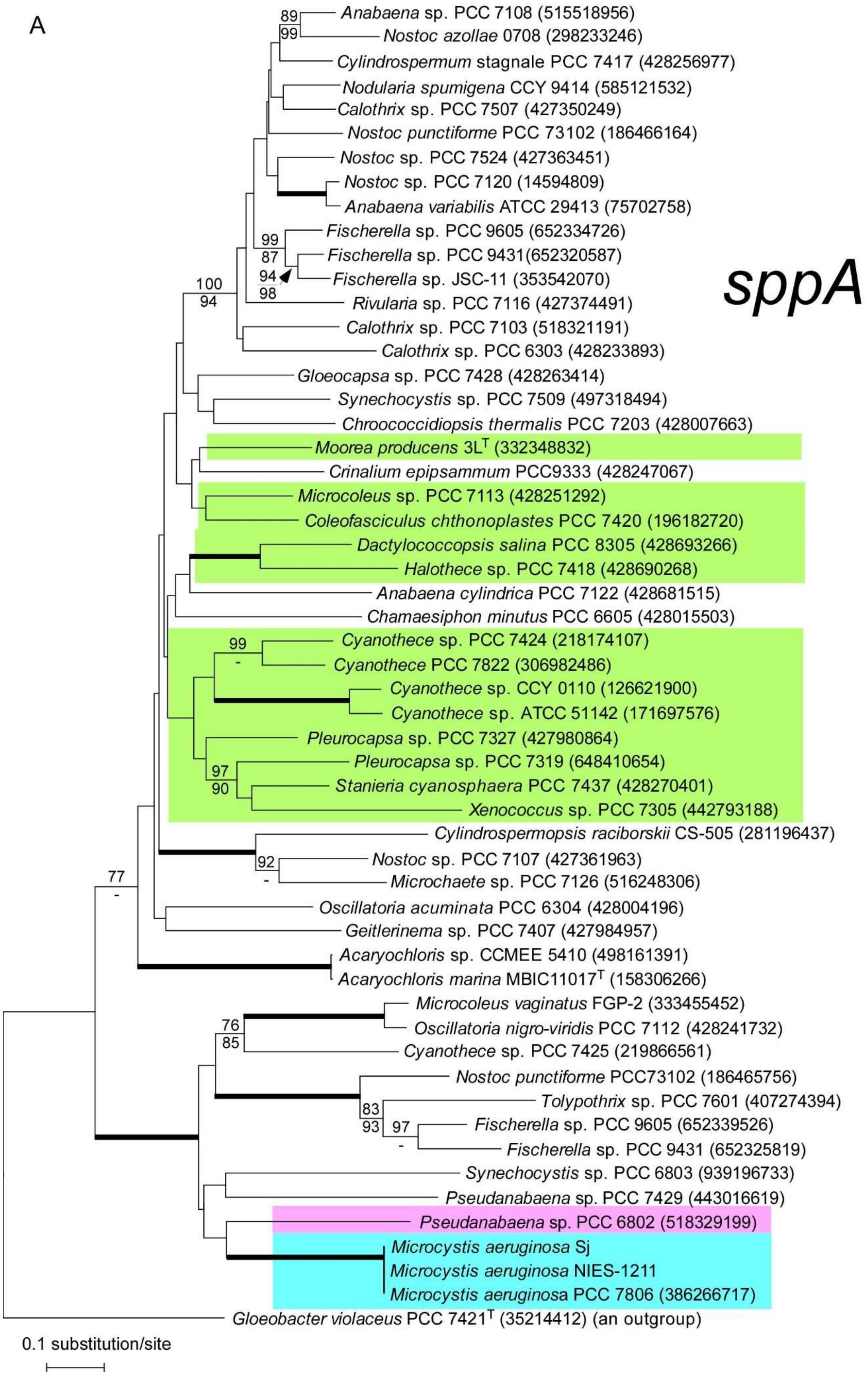

A
sppA
Anabaena sp. PCC 7108 (515518956)
Nostoc azollae 0708 (298233246)
Cylindrospermum stagnale PCC 7417 (428256977)
Nodularia spumigena CCY 9414 (585121532)
Calothrix sp. PCC 7507 (427350249)
Nostoc punctiforme PCC 73102 (186466164)
Nostoc sp. PCC 7524 (427363451)
Nostoc sp. PCC 7120 (14594809)
Anabaena variabilis ATCC 29413 (75702758)
Fischerella sp. PCC 9605 (652334726)
Fischerella sp. PCC 9431(652320587)
Fischerella sp. JSC-11 (353542070)
Rivularia sp. PCC 7116 (427374491)
Calothrix sp. PCC 7103 (518321191)
Calothrix sp. PCC 6303 (428233893)
Gloeocapsa sp. PCC 7428 (428263414)
Synechocystis sp. PCC 7509 (497318494)
Chroococcidiopsis thermalis PCC 7203 (428007663)
Moorea producens 3L^{T} (332348832)
Crinalium epipsammum PCC9333 (428247067)
Microcoleus sp. PCC 7113 (428251292)
Coleofasciculus chthonoplastes PCC 7420 (196182720)
Dactylococcopsis salina PCC 8305 (428693266)
Halothece sp. PCC 7418 (428690268)
Anabaena cylindrica PCC 7122 (428681515)
Chamaesiphon minutus PCC 6605 (428015503)
Cyanothece sp. PCC 7424 (218174107)
Cyanothece PCC 7822 (306982486)
Cyanothece sp. CCY 0110 (126621900)
Cyanothece sp. ATCC 51142 (171697576)
Pleurocapsa sp. PCC 7327 (427980864)
Pleurocapsa sp. PCC 7319 (648410654)
Stanieria cyanosphaera PCC 7437 (428270401)
Xenococcus sp. PCC 7305 (442793188)
Cylindrospermopsis raciborskii CS-505 (281196437)
Nostoc sp. PCC 7107 (427361963)
Microchaete sp. PCC 7126 (516248306)
Oscillatoria acuminata PCC 6304 (428004196)
Geitlerinema sp. PCC 7407 (427984957)
Acaryochloris sp. CCMEE 5410 (498161391)
Acaryochloris marina MBIC11017^{T} (158306266)
Microcoleus vaginatus FGP-2 (333455452)
Oscillatoria nigro-viridis PCC 7112 (428241732)
Cyanothece sp. PCC 7425 (219866561)
Nostoc punctiforme PCC73102 (186465756)
Tolypothrix sp. PCC 7601 (407274394)
Fischerella sp. PCC 9605 (652339526)
Fischerella sp. PCC 9431 (652325819)
Synechocystis sp. PCC 6803 (939196733)
Pseudanabaena sp. PCC 7429 (443016619)
Pseudanabaena sp. PCC 6802 (518329199)
Microcystis aeruginosa Sj
Microcystis aeruginosa NIES-1211
Microcystis aeruginosa PCC 7806 (386266717)
Gloeobacter violaceus PCC 7421^{T} (35214412) (an outgroup)
0.1 substitution/site

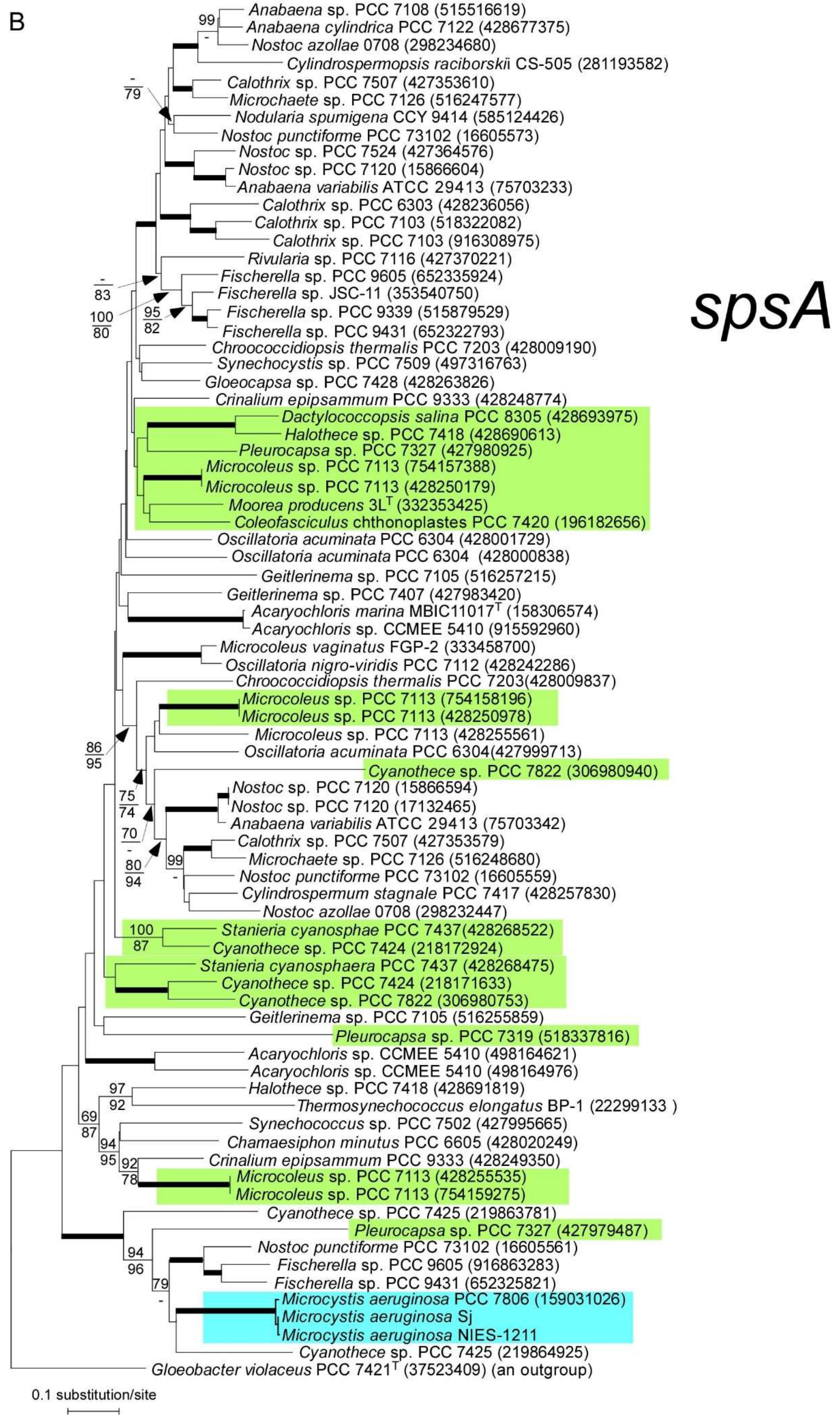

B
spsA
Anabaena sp. PCC 7108 (515516619)
Anabaena cylindrica PCC 7122 (428677375)
Nostoc azollae 0708 (298234680)
Cylindrospermopsis raciborskii CS-505 (281193582)
Calothrix sp. PCC 7507 (427353610)
Microchaete sp. PCC 7126 (516247577)
Nodularia spumigena CCY 9414 (585124426)
Nostoc punctiforme PCC 73102 (16605573)
Nostoc sp. PCC 7524 (427364576)
Nostoc sp. PCC 7120 (15866604)
Anabaena variabilis ATCC 29413 (75703233)
Calothrix sp. PCC 6303 (428236056)
Calothrix sp. PCC 7103 (518322082)
Calothrix sp. PCC 7103 (916308975)
Rivularia sp. PCC 7116 (427370221)
Fischerella sp. PCC 9605 (652335924)
Fischerella sp. JSC-11 (353540750)
Fischerella sp. PCC 9339 (515879529)
Fischerella sp. PCC 9431 (652322793)
Chroococcidiopsis thermalis PCC 7203 (428009190)
Synechocystis sp. PCC 7509 (497316763)
Gloeocapsa sp. PCC 7428 (428263826)
Crinalium epipsammum PCC 9333 (428248774)
Dactylococcopsis salina PCC 8305 (428693975)
Halothece sp. PCC 7418 (428690613)
Pleurocapsa sp. PCC 7327 (427980925)
Microcoleus sp. PCC 7113 (754157388)
Microcoleus sp. PCC 7113 (428250179)
Moorea producens 3L^T (332353425)
Coleofasciculus chthonoplastes PCC 7420 (196182656)
Oscillatoria acuminata PCC 6304 (428001729)
Oscillatoria acuminata PCC 6304 (428000838)
Geitlerinema sp. PCC 7105 (516257215)
Geitlerinema sp. PCC 7407 (427983420)
Acaryochloris marina MBIC11017^T (158306574)
Acaryochloris sp. CCMEE 5410 (915592960)
Microcoleus vaginatus FGP-2 (333458700)
Oscillatoria nigro-viridis PCC 7112 (428242286)
Chroococcidiopsis thermalis PCC 7203(428009837)
Microcoleus sp. PCC 7113 (754158196)
Microcoleus sp. PCC 7113 (428250978)
Microcoleus sp. PCC 7113 (428255561)
Oscillatoria acuminata PCC 6304(427999713)
Cyanothece sp. PCC 7822 (306980940)
Nostoc sp. PCC 7120 (15866594)
Nostoc sp. PCC 7120 (17132465)
Anabaena variabilis ATCC 29413 (75703342)
Calothrix sp. PCC 7507 (427353579)
Microchaete sp. PCC 7126 (516248680)
Nostoc punctiforme PCC 73102 (16605559)
Cylindrospermum stagnale PCC 7417 (428257830)
Nostoc azollae 0708 (298232447)
Stanieria cyanosphae PCC 7437(428268522)
Cyanothece sp. PCC 7424 (218172924)
Stanieria cyanosphaera PCC 7437 (428268475)
Cyanothece sp. PCC 7424 (218171633)
Cyanothece sp. PCC 7822 (306980753)
Geitlerinema sp. PCC 7105 (516255859)
Pleurocapsa sp. PCC 7319 (518337816)
Acaryochloris sp. CCMEE 5410 (498164621)
Acaryochloris sp. CCMEE 5410 (498164976)
Halothece sp. PCC 7418 (428691819)
Thermosynechococcus elongatus BP-1 (22299133)
Synechococcus sp. PCC 7502 (427995665)
Chamaesiphon minutus PCC 6605 (428020249)
Crinalium epipsammum PCC 9333 (428249350)
Microcoleus sp. PCC 7113 (428255535)
Microcoleus sp. PCC 7113 (754159275)
Cyanothece sp. PCC 7425 (219863781)
Pleurocapsa sp. PCC 7327 (427979487)
Nostoc punctiforme PCC 73102 (16605561)
Fischerella sp. PCC 9605 (916863283)
Fischerella sp. PCC 9431 (652325821)
Microcystis aeruginosa PCC 7806 (159031026)
Microcystis aeruginosa Sj
Microcystis aeruginosa NIES-1211
Cyanothece sp. PCC 7425 (219864925)
Gloeobacter violaceus PCC 7421^T (37523409) (an outgroup)
0.1 substitution/site

Supplementary Figure S5. Phylogenetic trees of sucrose genes. **A**, A NJ phylogenetic tree of *sppA*. **B,** A NJ phylogenetic tree of *spsA*. Bootstrap values (NJ/ML, >70) on the basis of 1 000 replicates are indicated at the respective nodes. Branches with >95 % NJ/ML bootstrap values are indicated in bold. Numbers in parentheses after the strain name indicate GenBank protein IDs. The color-coding is according to Fig 5**A**.
