# Supplementary material for: Adaptation of the Freshwater Bloom-Forming Cyanobacterium Microcystis aeruginosa to Brackish Water Is Driven by Recent Horizontal Transfer of Sucrose Genes
Source: Front Microbiol. 2018 Jun 5;9:1150. doi: 10.3389/fmicb.2018.01150 (PMC5996124; doi:10.3389/fmicb.2018.01150)
Supplement: Supplementary file 12 [file Image_6.PDF]

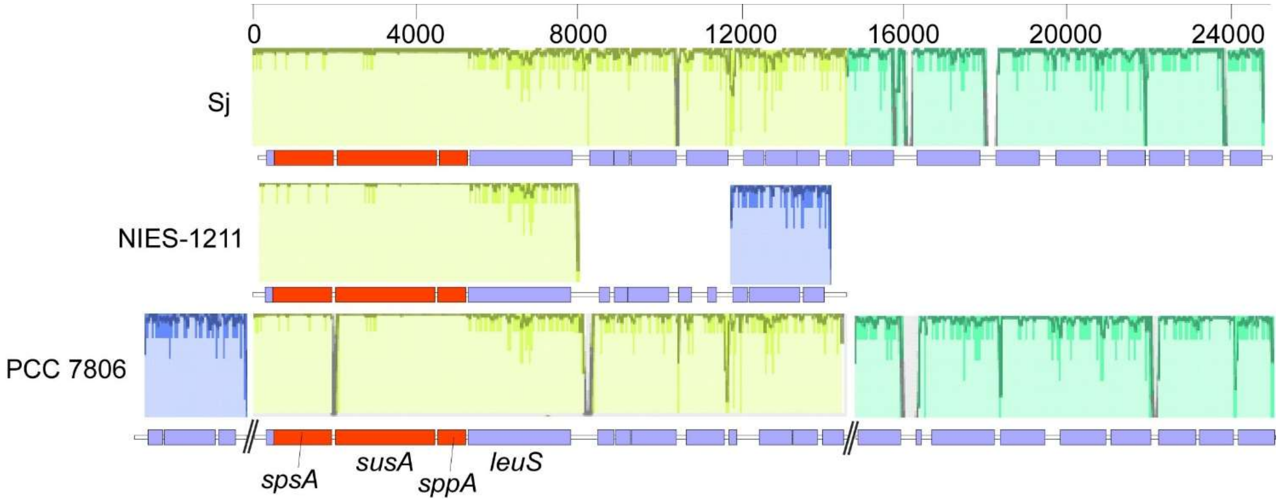

Supplementary Figure S6. Genomic organization and diversity of regions including sucrose genes. A Mauve alignment representation (Darling et al., 2004) of the contigs shows genetic diversity within regions. Vertical plot in the colored box indicates the average similarity in one region between two or three strains (deeper groove means higher dissimilarity). Regions in different colors indicate that the region is at a different location in the PCC 7806 genome. Note that much less genetic diversity is found within the sucrose gene cluster (highlighted in red) than in the surrounding regions. Open reading frames within 9 000 bps upstream of *sppA* in PCC 7806 have no orthologues in any contigs of Sj and NIES-1211.

Reference

Darling, A. C., Mau, B., Blattner, F. R., and Perna, N. T. (2004). Mauve: multiple alignment of conserved genomic sequence with rearrangements. *Genome Res.* 14, 1394–1403.
